# Design and synthesis of multi-target directed 1,2,3-triazole-dimethylaminoacryloyl-chromenone derivatives with potential use in Alzheimer's disease

**DOI:** 10.1186/s13065-020-00715-0

**Published:** 2020-10-27

**Authors:** Hajar Karimi Askarani, Aida Iraji, Arezoo Rastegari, Syed Nasir Abbas Bukhari, Omidreza Firuzi, Tahmineh Akbarzadeh, Mina Saeedi

**Affiliations:** 1 Department of Medicinal Chemistry, Faculty of Pharmacy, Tehran University of Medical Sciences, Tehran, Iran; 2 Medicinal and Natural Products Chemistry Research Center, Shiraz University of Medical Sciences, Shiraz, Iran; 3 Persian Medicine and Pharmacy Research Center, Tehran University of Medical Sciences, Tehran, Iran; 4 Department of Pharmaceutical Chemistry, College of Pharmacy, Aljouf University, Sakaka, 2014 Aljouf Saudi Arabia; 5 Medicinal Plants Research Center, Faculty of Pharmacy, Tehran University of Medical Sciences, Tehran, Iran

**Keywords:** Aβ aggregation, Biometal chelator, BuChE inhibitor, Chromenones, Neuroprotectivity, 1,2,3-triazole

## Abstract

To discover multifunctional agents for the treatment of Alzheimer's disease (AD), a new series of 1,2,3-triazole-chromenone derivatives were designed and synthesized based on the multi target-directed ligands approach. The in vitro biological activities included acetylcholinesterase (AChE) and butyrylcholinesterase (BuChE) inhibition as well as anti-Aβ aggregation, neuroprotective effects, and metal-chelating properties. The results indicated a highly selective BuChE inhibitory activity with an IC_50_ value of 21.71 μM for compound **10h** as the most potent compound. Besides, compound **10h** could inhibit self-induced Aβ_1–42_ aggregation and AChE-induced Aβ aggregation with 32.6% and 29.4% inhibition values, respectively. The Lineweaver–Burk plot and molecular modeling study showed that compound **10h** targeted both the catalytic active site (CAS) and peripheral anionic site (PAS) of BuChE. It should be noted that compound **10h** was able to chelate biometals. Thus, the designed scaffold could be considered as multifunctional agents in AD drug discovery developments. 
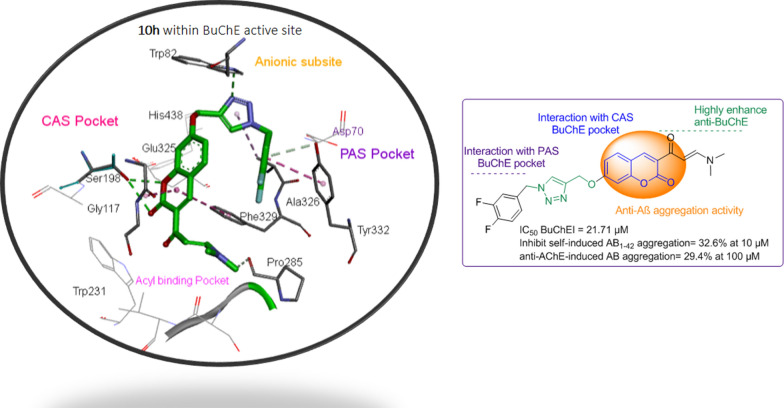

## Introduction

Dementia is one of the noteworthy problems in the public health management as over 80% of dementia cases are suffering from Alzheimer’s disease (AD). Currently, available therapies provide temporary symptomatic relief but do not target the distractive neuropathology. Therefore, a new treatment to delay or halt disease progression has remained as an urgent medical need.

The pathophysiological processes in AD have still remained unclear to this day. However, alongside its complexity, several neurodegenerative processes could be identified which include (I) aggregation of insoluble amyloid beta (Aβ) plaques mostly trigger from sequential cleavage of amyloid precursor protein (APP) by the aspartyl protease β-site APP cleaving enzyme-1 (BACE1) and γ-secretase, (II) neurofibrillary tangles (NFTs) form through hyperphosphorylation of tau proteins, (III) biometals dysfunction, and (IV) oxidative stress which in return results in synapse loss and death of neuronal cells in the brain [1]. Also, different hallmarks have been recognized including the loss of cholinergic neurons, reduction of the neurotransmitter acetylcholine (ACh), and increased expression of inflammatory mediators [2–4].

Based on the approved theory for AD, the loss of cholinergic neurons causes reduction of ACh. As a result, inhibition of the acetylcholinesterase (AChE) raises the level of ACh and improves cognitive performance at the early stage of AD. The critical point is that the AChE level decreases with the progression of AD, subsequently, AChE inhibition seems to be ineffective during the progression of AD [5, 6]. Interestingly, the level of butyrylcholinesterase (BuChE) remains unchanged or even increases at the late stage of disease [7]. BuChE can hydrolyze ACh and thereby, compensates the reduction of AChE activity [8]. A recent experiment with AChE knockout mice supported this hypothesis [9]. Results from further studies were in accordance with the role of BuChE in AD brains and showed a positive correlation between selective BuChE inhibition and improved cognitive performance and memory [10, 11].

BuChE has 65% amino acid sequence similarity to AChE along with mostly similar functions [12–14]. AChE and BuChE active sites have five subsites, including catalytic active site (CAS), peripheral anionic site (PAS), acyl binding pocket, oxyanion hole, and anionic subsite [15]. One of the structural differences between AChE and BuChE can be associated with acyl pocket size. More specifically, smaller residue such as Leu286 and Val288 of BuChE acyl pocket provide a larger site in BuChE while aromatic and bulky Phe295 and Phe297 residues of AChE acyl pocket afford smaller space in AChE. Mentioned structural differences contribute to the design of selective inhibitors [5, 8, 16].

Moreover, produced Aβ peptides can aggregate into Aβ plaques which initiate pathogenic cascade leading to neuronal loss and dementia. Inhibition of the accumulation of Aβ peptide in the brain could be another therapeutic strategy against the development of AD [2]. The metal chelatory potential of compounds has also been demonstrated to exert beneficial effects via decreasing plaque aggregation [17, 18].

## Results and discussion

### Design

**Fig. 1:**
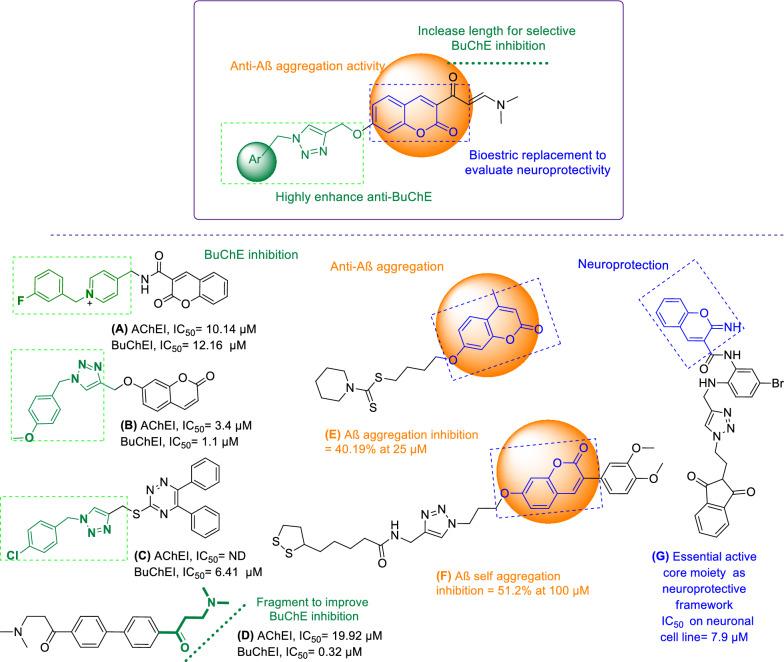
Designed hybrids investigated as selective BuChE inhibitors with anti-Aβ aggregatory, neuroprotective, and metal chelating properties

Because of the multifactorial and sporadic nature of AD, the modern approach “multi-target-one disease” could be efficient to develop effective agents to act simultaneously at different targets. Selective BuChE inhibitors could be a promising target for the treatment of AD at the moderate and advanced stages of the disease [19]. Closer looks at X-ray crystallography of BuChE depicted that it usually tolerates bigger scaffolds than AChE as the active site of BuChE is approximately 200 Å larger than AChE. Analysis of the potent BuChE inhibitors revealed that the N-containing ring could be effective for the interactions with ChE active site (Fig. 1). Molecular docking evaluation of compound **A** depicted that coumarin moiety interacted with Trp231 and Phe329 residues of CAS pocket and benzyl pyridinium moiety interacted with Trp82 of BuChE PAS pocket [20]. According to the interaction mode of compound **B,** it can be understood that 1,2,3-triazole-aryl moiety led to the formation of hydrophobic interactions with amino acids of PAS and chromenone ring oriented towards CAS pocket [21]. In the case of compound **C**, diphenyl fit into the BuChE CAS pocket while the benzyl triazine pendant group showed H-bond interaction with the PAS of BuChE [22, 23]. As appeared in compounds **B** and **C**, increasing bulkiness and length of drug candidate could increase the selectivity of BuChE over AChE. As can be seen in compound **D**, the presence of relatively spacious moieties can affect the interaction of the designed compounds with that amino acid within the active site of BuChE. As a result, introducing a dimethylamino propenone entity into our system increased the length and volume of the designed scaffold which might be a good strategy to increase selectivity towards BuChE [24]. Besides, this part could also exhibit metal-chelating potential [25].

In the case of anti-Aβ plaques aggregation, it is important to keep the potential moiety in the structure to inhibit the aggregation of the toxic peptide.

Coumarin structures as active natural compounds may simultaneously possess anti-oxidative [2, 26], neuroprotective [27] anti-ChE [20] and anti-Aβ aggregative properties [28, 29]. Coumarins pharmacophore owing to the presence of polar elements in the structure (Fig. 1, compound **E** and **F**) might help to inhibit amyloid fibril formation through the interaction with the polar surface of Aβ [30–32]. Hence, coumarins could serve as a potent framework for the prevention of Aβ_1-42_ aggregation [28]. In addition to the inhibition of BuChE and Aβ plaques aggregation, an inhibitor that can tackle toxicity of Aβ peptide, ROS and RNS could be effective for a longer period of AD progression. Recently, iminochromene ring was characterized as a potent neuroprotective agent. In this project, the iminochromene group of compound **G** was bioisosterically replaced with chromenone moiety to evaluate the possible neuroprotectivity [33]. Hence, in the present work, a molecular hybridization and bioisosteric replacement approaches were used to design and synthesize multi-target agents with anti-BuChE, anti-Aβ aggregation, neuroprotective, and metal chelating properties.

### Synthesis of 1,2,3-triazole-dimethylaminoacryloyl-chromenones

**Scheme 1.**
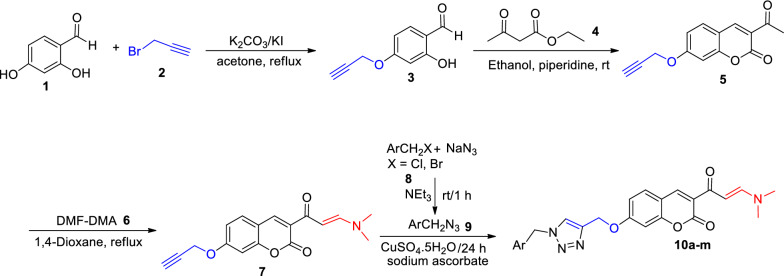
The synthetic route for the preparation of compounds **10a-m**

Synthesis of the tilted compounds **10a-m** was conducted according to the steps shown in Scheme 1. Desired starting material, 2-hydroxy-4-(prop-2-yn-1-yloxy)benzaldehyde (**3**) was exactly prepared according to the literature [34]. Then, the reaction of compound **3** and excess amounts of ethyl acetoacetate (**4**) in ethanol at room temperature overnight afforded 3-acetyl-7-(prop-2-yn-1-yloxy)-2*H*-chromen-2-one (**5**). Reaction of compound **5** and dimethylformamid-dimethylacetal (DMA-DMF) in 1,4-dioxane under reflux conditions for 6 h led to the formation of (*E*)-3-(3-(dimethylamino)acryloyl)-7-(prop-2-yn-1-yloxy)-2*H*-chromen-2-one (**7**). Finally, click reaction [35] of compound **7** and in situ prepared azides **9** in the presence of triethylamine and CuSO_4_.5H_2_O in the mixture of water and *tert*-butyl alcohol at room temperature for 24 h gave the corresponding products **10a**-**m**.

### AChE and BuChE inhibitory activity

In vitro anti-AChE and anti-BuChE activity of synthesized compounds, **10a**-**m** were performed based on the modified Ellman’s method [36] comparing with donepezil as the reference drug. Compounds were initially screened in vitro against AChE, and none of them exhibited inhibitory properties against the AChE enzyme. Interestingly, half of the 1,2,3-triazole- chromenone derivatives showed remarkable and selective inhibitory potency towards BuChE, which exerted a more prominent role at later stages of the disease [37].

As can be seen in Table 1, the BuChEI activity directly depended on the electronic property of substituents and their positions on the benzyl moiety connected to 1,2,3-triazole ring. Results showed that compound **10h** possessing 3,4-diF on the aryl ring induced the best BuChE inhibitory activity (IC_50_ = 21.71 μM); however, the elimination of 3-F completely changed the activity in such a manner that compound **10g** showed no inhibitory activity. *meta*-Fluorinated derivative **10f** was found to be a moderate inhibitor as the calculated IC_50_ value was 59.58 μM and *para*-fluorinated derivative **10g** showed no inhibitory activity towards BuChE (IC_50_ > 100 μM).

**Table 1:** Anti-cholinesterase activity of 1,2,3-triazole-dimethylaminoacryloyl-chromenone 10a-m 
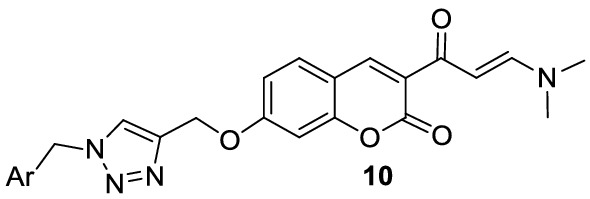

Entry	Ar	Product **10**	AChEI IC_50_ (µM)	BuChEI IC_50_ (µM)
1	C_6_H_5_	**10a**	> 100	34.41 ± 0.23
2	2-Me-C_6_H_4_	**10b**	> 100	35.73 ± 0.21
3	4-Me-C_6_H_4_	**10c**	> 100	> 100
4	3-MeO-C_6_H_4_	**10d**	> 100	23.44 ± 0.07
5	2-F-C_6_H_4_	**10e**	> 100	> 100
6	3-F-C_6_H_4_	**10f**	> 100	59.58 ± 0.05
7	4-F-C_6_H_4_	**10g**	> 100	> 100
8	3,4-diF-C_6_H_3_	**10h**	> 100	21.71 ± 0.57
9	2-Cl-C_6_H_4_	**10i**	> 100	> 100
10	4-Cl-C_6_H_4_	**10j**	> 100	> 100
11	2-Br-C_6_H_4_	**10k**	> 100	> 100
12	3-Br-C_6_H_4_	**10l**	> 100	65.96 ± 0.004
13	4-Br-C_6_H_4_	**10m**	> 100	> 100
14	**donepezil**		0.079 ± 0.002	5.19 ± 0.38

Data are expressed as mean ± SD (three independent experiments)

Considering the inhibitory activity of other halogenated derivatives **10i-m** depicted that chlorinated compounds **10i** and **10j** showed no inhibitory activity (IC_50_ > 100 μM). In the case of brominated derivatives **10k-m**, compounds **10l** possessing Br at 3-position of aryl ring showed moderate activity with IC_50_ value of 65.96 μM.

In the case of the electron-donating substituent (Me and OMe), relatively good results were obtained. The presence of methyl group at 2-position of aryl moiety (compound **10b**) led to relatively good anti-BuChE activity (IC_50_ = 35.73 μM); however, the presence of the same group at 4-position of compound **10c** completely diminished inhibitory activity (IC_50_ > 100 μM). Another point comes back to the derivative **10d** containing OMe group at 3-position on the benzyl ring which led to higher activity (IC_50_ = 23.44 μM). Finally, the absence of substituent on the aryl ring (compound **10a**) also depicted good BuChE inhibitory activity (IC_50_ = 34.41 μM).

The in vitro anti-ChE assay showed that the un-substituted benzyl derivative (**10a**) along with the *meta*-substituted analogs (**10d**, **10f**, and **10l**) had significant anti-BuChE activity. Introduction of extra small-size halogen groups such as F (compound **10h**) resulted in the most potent activity with an IC_50_ value of 21.71 µM.

### Kinetic study of BuChE inhibition

**Fig. 2:**
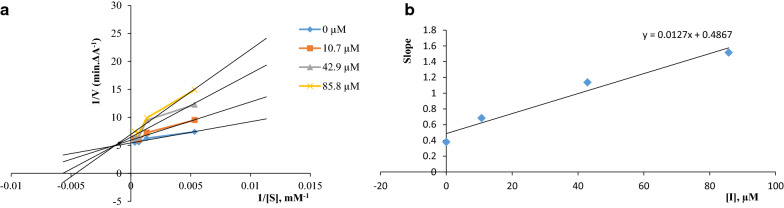
**a** Kinetic study of BuChE inhibition by compound **10h**. **b** Inhibition constant (*Ki*) of compound **10h**

The kinetic study was performed to investigate the mechanism of inhibition by compound **10h** against BuChE. Graphical analysis of the reciprocal Lineweaver–Burk plot of compound **10h** described a mixed-type inhibition pattern (Fig. 2a) in which compound **10h** may bound to the BuChE or it already bound to the substrate. Also, the *Ki* value was calculated using the secondary plot as 38.3 µM (Fig. 2b)

#### Inhibition of AChE-induced and self-induced Aβ aggregation

Aβ peptide is the major constituent of senile plaques in the brains of patients with AD. In this respect, the effect of the most potent compound **10h** was assessed for the inhibition against Aβ_1-42_ aggregation and AChE-induced Aβ_1-40_ peptide aggregation using the Thioflavin T (ThT) assay. Comparing with donepezil and tacrine as the reference compounds, it can be understood that **10h** was more potent than both controls in inhibiting Aβ_1-42_ self-aggregation, as depicted 32.6% inhibition at 10 µM (Table 2). Furthermore, compound **10h** inhibited AChE-induced Aβ aggregation by 29.4% at 100 μM.

**Table 2:** Inhibitory activities of compounds 10h against Aβ_1-42_ aggregation^a^

Samples	% Inhibition self-induced Aβ_1–42_ aggregation	% Inhibition AChE-induced Aβ aggregation
**10h**	32.6 ± 2.0 (10 µM) ^b^	29.4 ± 1.5 (100 µM) ^c^
Tacrine	7.6 ± 1.4 (10 µM)	6.7 ± 0.9 (100 µM)
Donepezil	18.1 ± 1.4 (10 µM)	25.2 ± 1.7 (100 µM)

^a^ Values are expressed as means ± SEM of three experiments. ^b^ Inhibition of self-induced Aβ_1-42_ aggregation (25 mM) produced by the test compound at 10 µM concentration. ^c^ Co-aggregation inhibition of Aβ_1-42_ and AChE (2 µM, ratio 100:1) by the test compound at 100 µM

### Neuroprotective studies on PC12 cell line

Compound **10h** was selected to study the neuroprotective ability using PC12 cell injury induced by Aβ_25–35_ by MTT assay. This compound depicted no neuroprotective effect on Aβ-induced on PC12 cells up to 50 μM. It can be understood that bioisosteric replacement of iminochromene moiety (compound **F,** Fig. 1) with chromenone ring did not induce the desired neuroprotectivity.

### Metal chelating

**Fig. 3:**
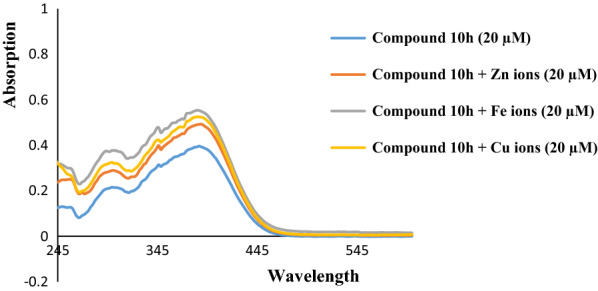
The absorbance change of compound **10h** alone and in the presence of Zn^2+^, Fe^2+^, and Cu^2+^ ions in the wavelength range from 200 to 600 nm. The experiments were performed in triplicate

Compound **10h** was tested for its metal chelating ability towards Fe^2+^, Cu^2+^, and Zn^2+^ ions (Fig. 3) The UV spectrum of methanolic solution (20 µM) of that compound showed two characteristic absorption peaks at 309.9 and 386.7 nm. After the interaction of compound **10h** with the above mentioned ions for 30 min, small shifts as well as absorption intensity changes were observed in the spectra confirming biometal-ligand interactions.

**Fig. 4:**
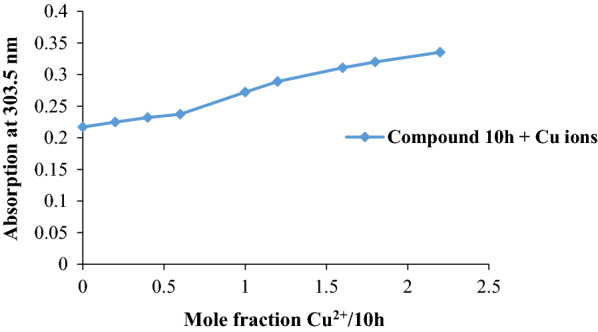
Determination of the stoichiometry of complex **10h**-Cu^+2^ using molar ratio method. The experiments were performed in triplicate

Interaction of compound **10h** with Zn^2+^ ions demonstrated two absorption peaks at 299.2 and 388.7 nm. Similar changes were observed in the case of Fe^2+^ ions and those absorptions were observed at 303.5 and 390.9 nm. When compound **10h** was treated with Cu^2+^ ions, two peaks were observed at 303.5 and 382.4 nm. The stoichiometry of complex **10h**-Cu^2+^ was also studied (Fig. 4) due to important role of copper ions in AD [38]. The concentration of the test compound **10h** was 20 μM and the final concentration of Cu^2+^ ranged from 0–44 μM with 4 μM intervals at 303.5 nm.

The plot was obtained by the corresponding absorption against the mole fraction of Cu^2+^ to ligand **10h**. According to the plot, the ratio 1:1 complexation ration of **10h**-Cu^2+^ can be seen at the fracture point of the plot with the mole fraction of 0.6.

### Docking study of BuChE

**Fig. 5:**
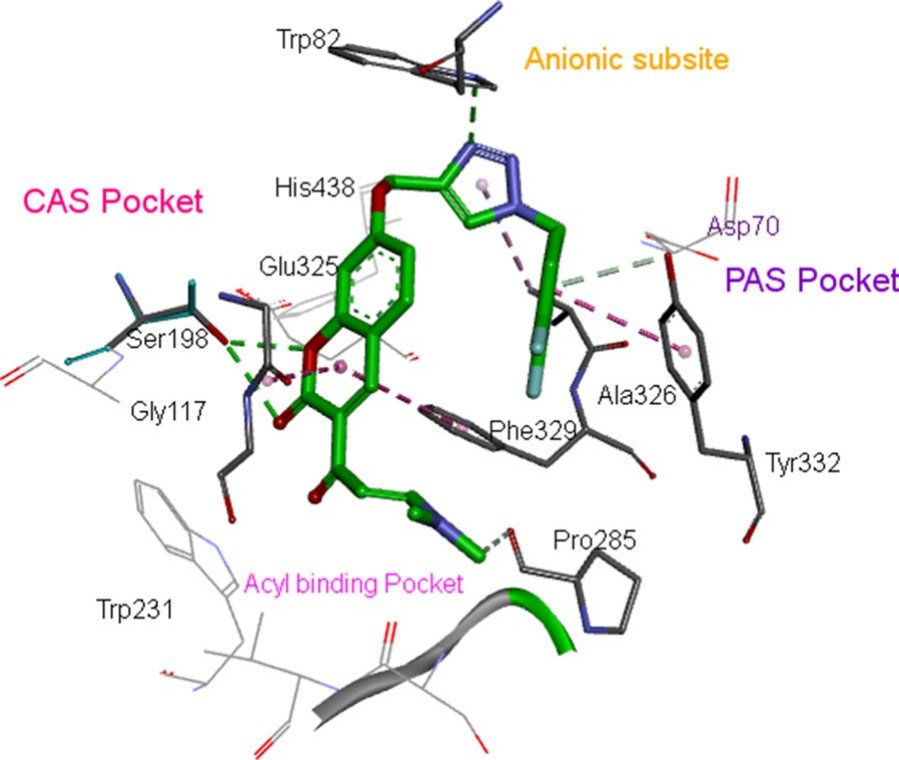
Schematic representation showing interactions of compound **10h** with the surrounding residues of BuChE (PDB code: 4BDS) [39]

As discussed in the introduction and designing sections, the volume of the BuChE active site is considerably bigger than the one found in the AChE, so BuChE can accommodate bulkier inhibitors, and this may constitute the basis for the selectivity of these derivatives. An overlay of the best pose for **10h** with BuChE was depicted in Fig. 5. Chromenenone core is mostly surrounded by residues of CAS pocket, while 3,4-difluoro benzyl moiety oriented towards PAS pocket. More specifically, the carbonyl group of the chromenenone ring form a hydrogen bond with the oxygen of Ser198 of the CAS while the another hydrogen bond interaction was seen between the oxygen of chromenenone pendant group and Ser198. Also, the 1,2,3-triazole ring formed a third hydrogen bond with Trp82 of the anionic subsite.

The nitrogen of the dimethylamino propenone interacted with Pro285 via Van der Waals interaction. The Van der Waals interaction was also constructed between 1,2,3-triazole moiety and Ala326 of the PAS residue. The 3,4-difluoro benzyl ring also showed π–π and van der Waals interactions with Tyr332. These results strongly supported the high potency of compound **10h** against BuChE.

It is concluded that not only dimethylamino propenone attached to the chromenone ring is an essential factor for designing an active and selective BuChE inhibitor, but also the nature of substituents on the aryl moieity connected to 1,2,3-triazole is the necessary element to afford a higher BuChE inhibitory effect. Further computer-aided lead optimization to improve anti-BuChE can be performed via replacing of the dimethylamino propenone pendant group with a cyclic amine to evaluate the size and bulkiness for the selective BuChE inhibition. Besides, the 1,2,3-triazole moiety can be substituted by different aliphatic spacer containing CO, NHCO, NH to improve the capability of H-bonding interaction with the active site.

### In silico ADME evaluation

In silico ADME/T studies of the synthesized compounds was performed using https://lmmd.ecust.edu.cn/admetsar2/ and https://preadmet.bmdrc.kr, to evaluate pharmacokinetic properties of possible and potential candidates/drug molecules which can be helpful for future anti-AD drug developments [40]. As shown in Table 3, most of the compounds showed drug-like characteristics based on Lipinski's rule of five (MW < 500, cLogP < 5, HB donor ≤ 5, HB acceptor ≤ 10). Our results indicated that lipophilicity and solubility of the derivatives were drug-like. Furthermore, molecular weight, cLogP, and blood–brain barrier were well within the standard ranges.

**Table 3:** Calculated molecular profile for synthesized compounds 10a-m

Compound	Descriptor						
	Mw	cLogP	H-bond acceptor	H-Bond Donor	BBB	Human intestinal absorption	Caco-2 permeability
**10a**	430.46	3.27	8	0	+ 0.9757	+ 0.9298	− 0.8029
**10b**	444.49	3.58	8	0	+ 0.9754	+ 0.9370	− 0.7985
**10c**	444.49	3.58	8	0	+ 0.9757	+ 0.9370	− 0.7908
**10d**	460.49	3.28	9	0	+ 0.9757	+ 0.9298	− 0.7587
**10e**	448.45	3.41	8	0	+ 0.9757	+ 0.9294	− 0.8157
**10f**	448.45	3.41	8	0	+ 0.9757	+ 0.9294	− 0.7970
**10g**	448.45	3.41	8	0	+ 0.9757	+ 0.9294	− 0.8152
**10h**	466.44	3.55	8	0	+ 0.9757	+ 0.9294	− 0.8211
**10i**	464.91	3.92	8	0	+ 0.9746	+ 0.9319	− 0.8289
**10j**	464.91	3.92	8	0	+ 0.9746	+ 0.9319	− 0.8211
**10k**	509.36	4.03	8	0	+ 0.9751	+ 0.9131	− 0.8299
**10l**	509.36	4.03	8	0	+ 0.9751	+ 0.9131	− 0.8115
**10m**	509.36	4.03	8	0	+ 0.9751	+ 0.9131	− 0.8306

## Materials and methods

### Instrumental methods

Melting points of synthesized compounds were determined on a Kofler hot stage apparatus. ^1^H and ^13^C NMR spectra were determined on a Varian FT-500, using TMS as an internal standard. IR spectra were recorded using KBr disks on a Bruker Tensor 27 FTIR spectrophotometer. Elemental analysis was carried out with an Elemental Analyzer system GmbH VarioEL CHN mode.

### Synthesis of 2-hydroxy-4-(prop-2-yn-1-yloxy)benzaldehyde (3)

Compound **3** was prepared from the reaction of 2,4-dihydroxybenzaldehyde **1** and propargyl bromide **2** in the presence of potassium carbonate (K_2_CO_3_) and potassium iodide (KI) in acetone at 50 ºC, according to the literature [34].

### Synthesis of 3-acetyl-7-(prop-2-yn-1-yloxy)-2H-chromen-2-one (5)

A few drops of piperidine were added to the mixture of compound **3** (1 mmol) and ethyl acetoacetate (2.5 mmol) **4** in ethanol (10 mL) and it was stirred overnight at room temperature to obtain yellow precipitates. After completion of the reaction (checked by TLC), they were filtered off and used for the next step with no further purification. It was also completely characterized and compared with reported in the literature [41].

### Synthesis of (E)-3-(3-(dimethylamino)acryloyl)-7-(prop-2-yn-1-yloxy)-2H-chromen-2-one (7)

The mixture of compound **5** (1 mmo l) and DMA-DMF **6** (2 mmol) in 1,4-dioxane (10 mL) was heated at reflux for 6 h. Then, the solvent was evaporated under vacuum and the residue was purified using plate chromatography with ethyl acetate as eluent.

Yield: 55%; M.p. 98–100 °C. IR (KBr): 2925, 2850, 2150, 1715, 1642, 1598 cm^−1^. ^1^H NMR (CDCl_3_, 500 MHz): δ = 8.58 (s, 1H, H4), 7.93 (d, *J* = 12.4 Hz, 1H, CH), 7.54 (d, *J* = 8.3 Hz, 1H, H5), 6.93–6.91 (m, 2H, H6, H8), 6.34 (d, *J* = 12.4 Hz, 1H, CH), 4.77 (s, 2H, CH_2_), 3.17 (s, 3H, CH_3_), 2.97 (s, 3H, CH_3_), 2.60 (1H, CH) ppm. ^13^C NMR (CDCl_3_, 125 MHz): 182.3, 162.1, 160.1, 156.4, 155.0, 145.9, 131.1, 123.3, 113.5, 113.4, 101.3, 95.2, 77.2, 77.0, 56.3, 45.2, 37.6 ppm.

### Synthesis of 1,2,3-triazole-dimethylaminoacryloyl-chromenone hybrids 10a-m

The final step was performed by the click reaction of compound **7** and in situ prepared azides **9**. For this purpose, a solution of benzyl chloride/bromide derivative **8** (1.1 mmol), sodium azide (0.06 g, 0.9 mmol), and triethylamine (0.13 g, 1.3 mmol) in water (4 mL) and *tert*-butyl alcohol (4 mL) was stirred at room temperature for 30 min. Then, compound **7** (0.5 mmol) and CuSO_4_.5H_2_O (7 mol%) were added to the mixture and it was continued for 24 h. Upon completion of the reaction checked TLC), the mixture was diluted with water, extracted with chloroform, and dried over anhydrous Na_2_SO_4_. After evaporation of the solvent, the residue was recrystallized from ethyl acetate and petroleum ether to give pure product **10**. In the case of some compounds, they were purified using plate chromatography with ethyl acetate as eluent.

### (E)-7-((1-Benzyl-1H-1,2,3-triazol-4-yl)methoxy)-3-(3-(dimethylamino)acryloyl)-2H-chromen-2-one (10a)

Yield: 54%; M.p. 186–188 °C. IR (KBr): 2920, 2853, 1715, 1640, 1597, 1558 cm^−1^. ^1^H NMR (CDCl_3_, 500 MHz): δ = 8.55 (s, 1H, triazole), 7.91 (d, *J* = 12.5 Hz, 1H, CH), 7.60 (s, 1H, H4), 7.50 (d, 1H, *J* = 8.5 Hz, H5), 7.38–7.29 (m, 5H, H2′, H3′, H4′, H5′, H6′), 6.92–6.90 (m, 2H, H6, H8), 6.33 (d, *J* = 12.5 Hz, 1H, CH), 5.55 (s, 2H, CH_2_), 5.24 (s, 2H, CH_2_), 3.17 (s, 3H, CH_3_), 2.96 (s, 3H, CH_3_) ppm. ^13^C NMR (CDCl_3_, 125 MHz): δ = 182.4, 171.7, 162.9, 156.2, 151.4, 145.6, 144.0, 134.7, 133.3, 131.1, 129.8, 128.9, 128.2, 122.6, 121.5, 113.3, 101.2, 92.2, 63.4, 57.2, 47.6, 38.1 ppm. Anal. calcd. for C_24_H_22_N_4_O_4_: C, 66.97; H, 5.15; N, 13.02. Found: C, 66.71; H, 5.38; N, 12.86.

### (E)-3-(3-(Dimethylamino)acryloyl)-7-((1-(2-methylbenzyl)-1H-1,2,3-triazol-4-yl)methoxy)-2H-chromen-2-one (10b)

Yield: 59%; M.p. 203–205 °C. IR (KBr): 2924, 2855, 1712, 1640, 1596, 1558 cm^−1^. ^1^H NMR (CDCl_3_, 500 MHz): δ = 8.58 (s, 1H, triazole), 7.92 (d, *J* = 12.3 Hz, 1H, CH), 7.52 (d, 1H, *J* = 8.3 Hz, H5), 7.46 (s, 1H, H4), 7.32–7.18 (m, 4H, H3′, H4′, H5′, H6′), 6.93–6.91 (m, 2H, H6, H8), 6.35 (d, *J* = 12.3 Hz, 1H, CH), 5.57 (s, 2H, CH_2_), 5.24 (s, 2H, CH_2_), 3.18 (s, 3H, CH_3_), 2.98 (s, 3H, CH_3_), 2.29 (s, 3H, CH_3_) ppm. ^13^C NMR (CDCl_3_, 125 MHz): δ = 182.4, 162.4, 160.2, 156.5, 154.9, 145.9, 143.0, 137.0, 132.0, 131.1, 130.7, 129.5, 129.3, 126.7, 123.5, 122.7, 113.3, 113.0, 101.3, 95.2, 62.4, 52.5, 45.3, 37.6, 19.0 ppm. Anal. calcd. for C_25_H_24_N_4_O_4_: C, 67.55; H, 5.44; N, 12.60. Found: C, 67.31; H, 5.70; N, 12.44.

### (E)-3-(3-(Dimethylamino)acryloyl)-7-((1-(4-methylbenzyl)-1H-1,2,3-triazol-4-yl)methoxy)-2H-chromen-2-one (10c)

Yield: 55%; M.p. 199–201 °C. IR (KBr): 2923, 2855, 1715, 1640, 1596, 1558 cm^−1^. ^1^H NMR (CDCl_3_, 500 MHz): δ = 8.57 (s, 1H, triazole), 7.92 (d, *J* = 12.3 Hz, 1H, CH), 7.55 (s, 1H, H4), 7.51 (d, 1H, *J* = 8.3 Hz, H5), 7.19–7.17 (m, 4H, H2′, H3′, H5′, H6′), 6.94–6.91 (m, 2H, H6, H8), 6.34 (d, *J* = 12.3 Hz, 1H, CH), 5.51 (s, 2H, CH_2_), 5.24 (s, 2H, CH_2_), 3.17 (s, 3H, CH_3_), 2.97 (s, 3H, CH_3_), 2.36 (s, 3H, CH_3_) ppm. ^13^C NMR (CDCl_3_, 125 MHz): δ = 182.5, 162.4, 160.2, 156.6, 1549, 145.9, 143.1, 138.9, 131.2, 130.7, 129.8, 128.2, 123.3, 122.8, 113.3, 133.2, 101.2, 95.2, 62.4, 54.6, 45.6, 37.6, 21.2 ppm. Anal. calcd. for C_25_H_24_N_4_O_4_: C, 67.55; H, 5.44; N, 12.60. Found: C, 67.40; H, 5.26; N, 12.71.

### (E)-3-(3-(Dimethylamino)acryloyl)-7-((1-(3-methoxybenzyl)-1H-1,2,3-triazol-4-yl)methoxy)-2H-chromen-2-one (10d)

Yield: 52%; M.p. 209–211 °C. IR (KBr): 2923, 2855, 1712, 1640, 1595, 1557 cm^−1^. ^1^H NMR (CDCl_3_, 500 MHz): δ = 8.57 (s, 1H, triazole), 7.92 (d, *J* = 12.4 Hz, 1H, CH), 7.59 (s, 1H, H4), 7.52 (d, 1H, *J* = 8.4 Hz, H5), 7.30 (t, *J* = 7.9 Hz, 1H, H5′), 6.94–6.81 (m, 5H, H6, H8, H2′, H4′, H6′), 6.35 (d, *J* = 12.4 Hz, 1H, CH), 5.52 (s, 2H, CH_2_), 5.25 (s, 2H, CH_2_), 3.79 (s, 3H, OCH_3_), 3.17 (s, 3H, CH_3_), 2.97 (s, 3H, CH_3_) ppm. ^13^C NMR (CDCl_3_, 125 MHz): δ = 182.4, 162.4, 160.2, 156.6, 154.9, 145.9, 144.0, 143.2, 135.7, 130.7, 130.3, 123.4, 122.9, 119.3, 114.3, 113.8, 133.3, 112.5, 101.2, 95.2, 62.4, 55.3, 54.3, 45.2, 37.6 ppm. Anal. calcd. for C_25_H_24_N_4_O_5_: C, 65.21; H, 5.25; N, 12.17. Found: C, 65.41; H, 5.10; N, 12.32.

### (E)-3-(3-(Dimethylamino)acryloyl)-7-((1-(2-fluorobenzyl)-1H-1,2,3-triazol-4-yl)methoxy)-2H-chromen-2-one (10e)

Yield: 55%; M.p. 180–183 °C. IR (KBr): 2924, 2852, 1712, 1640, 1597, 1558 cm^−1^. ^1^H NMR (CDCl_3_, 500 MHz): δ = 8.57 (s, 1H, triazole), 7.91 (d, *J* = 12.4 Hz, 1H, CH), 7.67 (s, 1H, H4), 7.51 (d, 1H, *J* = 8.0 Hz, H5), 7.39–7.38 (m, 1H, H4′), 7.30 (td, *J* = 7.6, 1.5 Hz, 1H, H3′), 7.17–7.11 (m, 2H, H5′, H6′), 6.94–6.92 (m, 2H, H6, H8), 6.34 (d, *J* = 12.4 Hz, 1H, CH), 5.61 (s, 2H, CH_2_), 5.25 (s, 2H, CH_2_), 3.17 (s, 3H, CH_3_), 2.97 (s, 3H, CH_3_) ppm. ^13^C NMR (CDCl_3_, 125 MHz): δ = 182.3, 162.4, 160.6 (d, *J*_*C-F*_ = 251.2 Hz), 160.1, 158.7, 156.6, 154.8, 145.9, 143.2, 131.1 (d, *J*_*C-F*_ = 8.1 Hz), 130.7 (d, *J*_*C-F*_ = 3.3 Hz), 124.9 (d, *J*_*C-F*_ = 3.4 Hz), 123.3, 121.3, 121.6 (d, *J*_*C-F*_ = 14.5 Hz), 115.9 (d, *J*_*C-F*_ = 21.03 Hz), 113.3, 113.2, 101.2, 95.2, 62.4, 47.9, 45.2, 37.5 ppm. Anal. calcd. for C_24_H_21_FN_4_O_4_: C, 64.28; H, 4.72; N, 12.49. Found: C, 64.36; H, 4.57; N, 12.55.

### (E)-3-(3-(Dimethylamino)acryloyl)-7-((1-(3-fluorobenzyl)-1H-1,2,3-triazol-4-yl)methoxy)-2H-chromen-2-one (10f)

Yield: 60%; M.p. 183–185 °C. IR (KBr): 2922, 2850, 1713, 1640, 1597 cm^−1^. ^1^H NMR (CDCl_3_, 500 MHz): δ = 8.57 (s, 1H, triazole), 7.91 (d, *J* = 12.3 Hz, 1H, CH), 7.61 (s, 1H, H4), 7.51 (d, 1H, *J* = 8.3 Hz, H5), 7.36 (td, 1H, *J* = 7.5, 2.2 Hz, H5′), 7.08–7.04 (m, 2H, H4′, H6′), 6.97 (d, 1H, *J* = 9.2 Hz, H2′), 6.94–6.91 (m, 2H, H6, H8), 6.34 (d, *J* = 12.3 Hz, 1H, CH), 5.55 (s, 2H, CH_2_), 5.26 (s, 2H, CH_2_), 3.17 (s, 3H, CH_3_), 2.96 (s, 3H, CH_3_) ppm. ^13^C NMR (CDCl_3_, 125 MHz): δ = 180.0, 162.3, 160.7 (d, *J*_*C-F*_ = 262.2 Hz), 160.1, 156.7, 154.9, 145.8, 143.5, 136.6 (d, *J*_*C-F*_ = 19.4 Hz), 130.8 (d, *J*_*C-F*_ = 8.1 Hz), 130.7, 123.6, 123.3, 122.9, 115.9 (d, *J*_*C-F*_ = 20.9 Hz), 115.1 (d, *J*_*C-F*_ = 22.0 Hz), 113.3, 113.2, 101.2, 95.2, 62.4, 53.7, 45.0, 37.6 ppm. Anal. calcd. for C_24_H_21_FN_4_O_4_: C, 64.28; H, 4.72; N, 12.49. Found: C, 64.47; H, 4.84; N, 12.60.

### (E)-3-(3-(Dimethylamino)acryloyl)-7-((1-(4-fluorobenzyl)-1H-1,2,3-triazol-4-yl)methoxy)-2H-chromen-2-one (10g)

Yield: 62%; M.p. 183–185 °C. IR (KBr): 2923, 2855, 1714, 1640, 1598 cm^−1^. ^1^H NMR (CDCl_3_, 500 MHz): δ = 8.56 (s, 1H, triazole), 7.92 (d, *J* = 12.4 Hz, 1H, CH), 7.58 (s, 1H, H4), 7.51 (d, 1H, *J* = 8.4 Hz, H5), 7.29 (dd, *J* = 8.7, 5.1 Hz, 2H, H2′, H6′), 7.07 (t, *J* = 8.7 Hz, 2H, H3′, H5′), 6.93–6.91 (m, 2H, H6, H8), 6.33 (d, *J* = 12.4 Hz, 1H, CH), 5.52 (s, 2H, CH_2_), 5.24 (s, 2H, CH_2_), 3.16 (s, 3H, CH_3_), 2.96 (s, 3H, CH_3_) ppm. ^13^C NMR (CDCl_3_, 125 MHz): δ = 180.1, 162.4, 160.5 (d, *J*_*C-F*_ = 260.5 Hz), 160.1, 156.5, 154.8, 145.1, 143.4, 134.5 (d, *J*_*C-F*_ = 19.5 Hz), 130.7, 130.1, 123.3, 122.8, 116.2 (d, *J*_*C-F*_ = 21.0 Hz), 113.3, 113.2, 101.2, 95.1, 62.1, 53.5, 45.0, 37.5 ppm. Anal. calcd. for C_24_H_21_FN_4_O_4_: C, 64.28; H, 4.72; N, 12.49. Found: C, 64.44; H, 4.60; N, 12.27.

### (E)-7-((1-(3,4-Difluorobenzyl)-1H-1,2,3-triazol-4-yl)methoxy)-3-(3-(dimethylamino)acryloyl)-2H-chromen-2-one (10h)

Yield: 59%; M.p. 188–190 °C. IR (KBr): 2922, 2850, 1718, 1640, 1594 cm^−1^. ^1^H NMR (CDCl_3_, 500 MHz): δ = 8.57 (s, 1H, triazole), 7.91 (d, *J* = 12.3 Hz, 1H, CH), 7.63 (s, 1H, H4), 7.52 (d, 1H, *J* = 8.4 Hz, H5), 7.21–7.11 (m, 2H, H5′, H6′), 7.06–7.04 (m, 1H, H2′), 6.93–6.91 (m, 2H, H6, H8), 6.33 (d, *J* = 12.3 Hz, 1H, CH), 5.52 (s, 2H, CH_2_), 5.27 (s, 2H, CH_2_), 3.18 (s, 3H, CH_3_), 2.97 (s, 3H, CH_3_) ppm. ^13^C NMR (CDCl_3_, 125 MHz): δ = 182.3, 160.1, 156.6, 154.9, 150.5 (d, *J*_*C-F*_ = 243.1 Hz), 149.6, (d, *J*_*C-F*_ = 240.0 Hz), 145.8, 144.0, 143.6, 132.8, 131.2, 130.7, 124.3, 123.4, 122.9, 118.1 (d, *J*_*C-F*_ = 17.2), 117.3 (d, *J*_*C-F*_ = 17.6), 113.3, 101.2, 95.2, 62.4, 53.2, 45.2, 37.6 ppm. Anal. calcd. for C_24_H_20_F_2_N_4_O_4_: C, 61.80; H, 4.32; N, 12.01. Found: C, 61.63; H, 4.17; N, 11.84.

### (E)-7-((1-(2-Chlorobenzyl)-1H-1,2,3-triazol-4-yl)methoxy)-3-(3-(dimethylamino)acryloyl)-2H-chromen-2-one (10i)

Yield: 58%; M.p. 178–180 °C. IR (KBr): 2923, 2852, 1713, 1640, 1597 cm^−1^. ^1^H NMR (CDCl_3_, 500 MHz): δ = 8.60 (s, 1H, triazole), 7.91 (d, *J* = 11.0 Hz, 1H, CH), 7.68 (s, 1H, H4), 7.51 (d, 1H, *J* = 8.1 Hz, H5), 7.43 (d, 1H, *J* = 7.9 Hz, H3′), 7.32–7.24 (m, 3H, H4′, H5′, H6′), 6.93–6.87 (m, 2H, H6, H8), 6.33 (d, *J* = 11.0 Hz, 1H, CH), 5.69 (s, 2H, CH_2_), 5.29 (s, 2H, CH_2_), 3.16 (s, 3H, CH_3_), 2.96 (s, 3H, CH_3_) ppm. ^13^C NMR (CDCl_3_, 125 MHz): δ = 182.7, 171.5, 163.8, 158.5, 156.3, 151.7, 144.5, 133.6, 131.7, 130.7, 130.5, 130.4, 130.0, 127.7, 123.4, 120.1, 113.3, 112.8, 100.6, 96.6, 61.1, 52.3, 46.4, 40.4 ppm. Anal. calcd. for C_24_H_21_ClN_4_O_4_: C, 62.00; H, 4.55; N, 12.05. Found: C, 61.81; H, 4.38; N, 11.90.

### (E)-7-((1-(4-Chlorobenzyl)-1H-1,2,3-triazol-4-yl)methoxy)-3-(3-(dimethylamino)acryloyl)-2H-chromen-2-one (10j)

Yield: 61%; M.p. 178–180 °C. IR (KBr): 2891, 2850, 1715, 1640, 1597, 1558 cm^−1^. ^1^H NMR (CDCl_3_, 500 MHz): δ = 8.54 (s, 1H, triazole), 7.90 (d, *J* = 12.4 Hz, 1H, CH), 7.61 (s, 1H, H4), 7.50 (d, *J* = 8.5 Hz, 1H, H5), 7.34 (d, *J* = 8.1 Hz, 2H, H3′, H5′), 7.22 (d, *J* = 8.1 Hz, 2H, H2′, H6′),

6.92–6.89 (m, 2H, H6, H8), 6.34 (d, *J* = 12.4 Hz, 1H, CH), 5.52 (s, 2H, CH_2_), 5.19 (s, 2H, CH_2_), 3.11 (s, 3H, CH_3_), 2.95 (s, 3H, CH_3_) ppm. ^13^C NMR (CDCl_3_, 125 MHz): δ = 182.9, 162.4, 160.1, 156.5, 154.9, 145.8, 144.2, 143.4, 134.9, 130.7, 129.5, 129.4, 123.3, 122.9, 113.3, 133.2, 101.2, 95.2, 62.4, 53.5, 45.2, 37.6 ppm. Anal. calcd. for C_24_H_21_ClN_4_O_4_: C, 62.00; H, 4.55; N, 12.05. Found: C, 62.11; H, 4.40; N, 12.28.

### (E)-7-((1-(2-Bromobenzyl)-1H-1,2,3-triazol-4-yl)methoxy)-3-(3-(dimethylamino)acryloyl)-2H-chromen-2-one (10k)

Yield: 58%; M.p. 202–205 °C. IR (KBr): 2924, 2850, 1709, 1641, 1598, 1559 cm^−1^. ^1^H NMR (CDCl_3_, 500 MHz): δ = 8.57 (s, 1H, triazole), 7.92 (d, *J* = 12.3 Hz, 1H, CH), 7.70 (s, 1H, H4), 7.63 (d, 1H, *J* = 7.5 Hz, H3′), 7.52 (d, 1H, *J* = 8.3 Hz, H5), 7.33 (t, 1H, *J* = 7.5 Hz, H5′), 7.25–7.21 (m, 2H, H4′, H6′), 6.95–6.93 (m, 2H, H6, H8), 6.34 (d, *J* = 12.3 Hz, 1H, CH), 5.69 (s, 2H, CH_2_), 5.27 (s, 2H, CH_2_), 3.21 (s, 3H, CH_3_), 2.97 (s, 3H, CH_3_) ppm. ^13^C NMR (CDCl_3_, 125 MHz): δ = 182.5, 162.4, 160.1, 156.6, 154.9, 145.9, 143.1, 133.8, 133.4, 133.3, 130.7, 130.6, 130.5, 128.3, 123.6, 123.3, 113.4, 113.3, 100.4, 95.2, 62.0, 54.0, 44.8, 37.2 ppm. Anal. calcd. for C_24_H_21_BrN_4_O_4_: C, 56.59; H, 4.16; N, 11.00. Found: C, 56.37; H, 4.30; N, 11.21.

### (E)-7-((1-(3-Bromobenzyl)-1H-1,2,3-triazol-4-yl)methoxy)-3-(3-(dimethylamino)acryloyl)-2H-chromen-2-one (10l)

Yield: 64%; M.p. 218–220 °C. IR (KBr): 2925, 2856, 1710, 1640, 1595 cm^−1^. ^1^H NMR (CDCl_3_, 500 MHz): δ = 8.59 (s, 1H, triazole), 7.93 (d, *J* = 12.4 Hz, 1H, CH), 7.61 (s, 1H, H4), 7.55–7.51 (m, 3H, H5, H2′, H4′), 7.26–7.18 (m, 2H, H5′, H6′), 6.96–6.94 (m, 2H, H6, H8), 6.35 (d, *J* = 12.4 Hz, 1H, CH), 5.51 (s, 2H, CH_2_), 5.28 (s, 2H, CH_2_), 3.19 (s, 3H, CH_3_), 2.98 (s, 3H, CH_3_) ppm. ^13^C NMR (CDCl_3_, 125 MHz): δ = 180.1, 162.3, 160.2, 156.5, 154.9, 145.9, 143.5, 136.4, 133.7, 132.1, 131.1, 130.9, 130.8, 126.6, 122.9, 122.2, 113.4, 113.3, 101.1, 95.0, 62.8, 53.6, 44.8, 38.8 ppm. Anal. calcd. for C_24_H_21_BrN_4_O_4_: C, 56.59; H, 4.16; N, 11.00. Found: C, 56.31; H, 4.24; N, 11.18.

### (E)-7-((1-(4-Bromobenzyl)-1H-1,2,3-triazol-4-yl)methoxy)-3-(3-(dimethylamino)acryloyl)-2H-chromen-2-one (10m)

Yield: 64%; M.p. 173–175 °C. IR (KBr): 2925, 2850, 1715, 1640, 1597 cm^−1^. ^1^H NMR (CDCl_3_, 500 MHz): δ = 8.57 (s, 1H, triazole), 7.91 (d, *J* = 12.3 Hz, 1H, CH), 7.60 (s, 1H, H4), 7.52–7.51 (m, 3H, H5, H3′, H5′), 7.17 (d, *J* = 8.4 Hz, 2H, H2′, H6′), 6.93–6.91 (m, 2H, H6, H8), 6.34 (d, *J* = 12.3 Hz, 1H, CH), 5.51 (s, 2H, CH_2_), 5.22 (s, 2H, CH_2_), 3.17 (s, 3H, CH_3_), 2.96 (s, 3H, CH_3_) ppm. ^13^C NMR (CDCl_3_, 125 MHz): δ = 182.4, 162.9, 160.1, 156.6, 154.9, 146.6, 144.4, 143.5, 133.3, 132.4, 130.7, 129.8, 123.3, 123.1, 122.9, 112.7, 100.8, 95.2, 62.4, 53.6, 45.2, 37.6 ppm. Anal. calcd. for C_24_H_21_BrN_4_O_4_: C, 56.59; H, 4.16; N, 11.00. Found: C, 56.68; H, 4.38; N, 10.87 (Additional file 1).

### Inhibitory activities against AChE and BuChE

All enzymes and reagents required for the assay were obtained from Aldrich. The in vitro anticholinesterase activity of all synthesized compounds **10a-m** was assayed using modified Ellman's method using a 96-well plate reader (BioTek ELx808) according to the literature [36, 42]. Initially, the stock solutions of compounds **10** were prepared by dissolving the test compound (1 mg) in DMSO (1 mL) and then, diluted solutions at final concentrations of 1, 10, 20, and 40 μg/mL were prepared using methanol. Each well contained 50 µL potassium phosphate buffer (KH_2_PO_4_/ K_2_HPO_4_, 0.1 M, pH 8), 25 µL sample solution, and 25 µL enzyme (final concentration 0.22 U/mL in buffer). Control experiments were also performed under the same conditions without enzyme. After incubation at room temperature for 15 min, 125 µL DTNB (3 mM in buffer) was added and the characterization of enzymatic reaction was spectrometrically performed at 405 nm followed by the addition of substrate (ATCI 3 mM in water) after 5–10 min. The IC_50_ values were determined graphically from inhibition curves (log inhibitor concentration vs. percent of inhibition). Also, the same method was used for the BuChE inhibition assay.

### Kinetic characterization of BuChE inhibitory activity

The kinetic study for the inhibition of BuChE by compound **10h** was carried out according to Ellman's method used for the inhibition assay using four different concentrations of inhibitor (0, 10.7, 42.9, and 85.8 µM). The Lineweaver–Burk reciprocal plot was provided by plotting 1/V against 1/[S] at variable concentrations of butyrylthiocholine as the substrate (187.5, 750, 1500, 3000 µM). The inhibition constant *Ki* was achieved by the plot of slopes versus the corresponding concentrations of compound **10h** [43, 44].

### Inhibition of Aβ_1-42 _aggregation and disaggregation of aggregated Aβ_1-40_ induced by AChE

Inhibition of Aβ1-42 self-aggregation was measured by ThT fluorescence assay. The details of the method were reported in our previous study [45]. To study Aβ_42_ aggregation inhibition, a reported method, based on the fluorescence emission of ThT was followed. Briefly, the mixtures of Aβ_1-40_ peptide (Bachem company, Switzerland) and AChE (Sigma, Electrophorus electricus), in the presence or absence of the test inhibitor were incubated for 24 h at room temperature. The final concentrations of Aβ (dissolved in DMSO and diluted 0.215 M sodium phosphate buffer, pH 8), AChE (dissolved in 0.215 M sodium phosphate buffer, pH 8.0), and the test compound are 200 µM, 2 µM and 100 µM, respectively. After co-incubation, 20 µL of the mixed solution was diluted to a final volume of 2 mL with ThT (1.5 µM in 50 mM glycine–NaOH buffer, pH 8.5) and the absorbance was measured with a multi-mode plate reader at the excitation and emission wavelength of λex = 450 nm and λem = 485 nm, respectively [45].

### Neuroprotection assay against Aβ-induced damage

MTT reduction assay was used to evaluate the neuroprotective effect of compound **10h** on neuronal PC12 cell damage induced by Aβ_25-35_. The cells were grown in monolayer culture on collagen-coated plates at 37 °C in a humidified atmosphere of 5% CO_2_. Neuronal PC12 cells were plated at a density of 5 × 10^5^ cells/well on 96-well plates. The cells were pre-incubated with compound **10h** for 3 h before human Aβ _25–35_ (final concentration of 5 μM) was added. After 24 h, 90 μL the medium was taken out and 20 μL of MTT (0.5 mg/ml dissolved in RPMI containing phenol red) was added and incubated for an additional 2 h at 37 °C. The absorbance (A570 nm) was measured using a Bio-Rad microplate reader (Model 680, Bio-Rad). The details were reported in our previous work [33, 46].

### Metal chelation studies

To study the metal chelating ability, the solutions of compound **10h** and Fe^2+^, Cu^2+^, and Zn^2+^ ions (from FeSO_4_, CuCl_2_.2H_2_O, and ZnCl_2_) were prepared in methanol. The mixture of compound **10h** (1 mL) and the test ion solutions (1 mL) with the same final concentration of 20 µM in a 1 cm quartz cuvette was incubated at room temperature for 30 min. Then, the absorption spectra were recorded in the range of 200–600 nm.

The stoichiometry of complex **10h**-Cu^2+^ was also studied using the molar ratio method [47, 48]. The concentration of compound **10h** was 20 μM and the final concentration of Cu^2+^ ranged from 0–44 μM with 4 μM intervals at 303.5 nm. The plot was obtained by the corresponding absorption versus mole fraction of Cu^2+^ to ligand **10h**. All experiments were performed in triplicate.

### Molecular docking

The molecular docking studies of the most potential ligand was performed on BuChE (PDB code: 4BDS) [39] to observe the binding orientation and consensual binding interactions using AutoDock 4.2. The X-ray crystal structure of the receptor was downloaded from the PDB database. All water and ligand molecules were removed from the structure, and the protein was prepared for docking. The co-crystallized ligand within the PDB structures was defined as a center of the binding site. All ligands were created using Chem3D Ultra software, and energy minimizations were done by the semiempirical MM^+^ [49]. The compounds were docked into the active site of proteins using default parameters for each ligand with 100 runs and 27,000 as the maximum number of generations. The grid boxes were set with 60, 60, and 60 points in the x, y, and z directions, respectively. All other options were set as default. The calculated geometries were ranked in terms of free energy of binding and the best pose was selected for further analysis. Molecular visualizations were performed by Discovery Studio 4.0 client software [20].

### Prediction of ADME descriptors

ADME-Tox properties of the synthesized compounds were performed by using online servers especially https://lmmd.ecust.edu.cn:8000/predict/ and https://preadmet.bmdrc.kr.

## Conclusion

In summary, a new series of 1,2,3-triazole-dimethylaminoacryloyl-chromenone derivatives were designed and synthesized as multifunctional anti-Alzheimer’s agents. All the target compounds were synthesized and screened as AChE/BuChE inhibitors. The most active compound was further evaluated by the multiple biological activities including Aβ_1-42_ aggregation inhibition, metal-chelating properties, and neuroprotective effects against Aβ_25-35_-induced PC12 cell injury. Our results showed that these compounds had a high inhibitory potency and selectivity towards BuChE with an IC_50_ value of 21.71 μM for **10h** as the most potent BuChE inhibitor. The inhibition kinetic analysis revealed a mixed-type inhibition pattern for this compound. The molecular modeling study of the most potent compound **10h** with BuChE indicated that it bound to both CAS and PAS of the BuChE. Moreover, this compound had a significant anti-Aβ aggregation capacity and served as a metal chelator. These results indicated that this hybridization approach could be a successful strategy for the further developments of potential multifunctional candidates against AD.

## Supplementary information

**Additional file 1.** The supplementary file include copies of NMR spectra and elemental analysis report.

## Data Availability

The datasets used and analyzed during the current study are available from the corresponding author on reasonable request.

## Abbreviations

AD: Alzheimer's disease
AChE: Acetylcholinesterase
BuChE: Butylcholinesterase
CAS: Catalytic active site
PAS: Peripheral anionic site
Aβ: Amyloid βeta
APP: Amyloid precursor protein
BACE1: β-Site APP cleaving enzyme-1
NFTs: Neurofibrillary tangles
IC_50_: The half maximal inhibitory concentration
^1^H NMR: Proton nuclear magnetic resonance
^13^C NMR: Carbon-13 nuclear magnetic resonance
