# Supplementary material for: Design and synthesis of multi-target directed 1,2,3-triazole-dimethylaminoacryloyl-chromenone derivatives with potential use in Alzheimer's disease
Source: BMC Chem. 2020 Oct 27;14(1):64. doi: 10.1186/s13065-020-00715-0 (PMC7592376; doi:10.1186/s13065-020-00715-0)

***(E)-3-(3-(Dimethylamino)acryloyl)-7-(prop-2-yn-1-yloxy)-2H-chromen-2-one (7)***


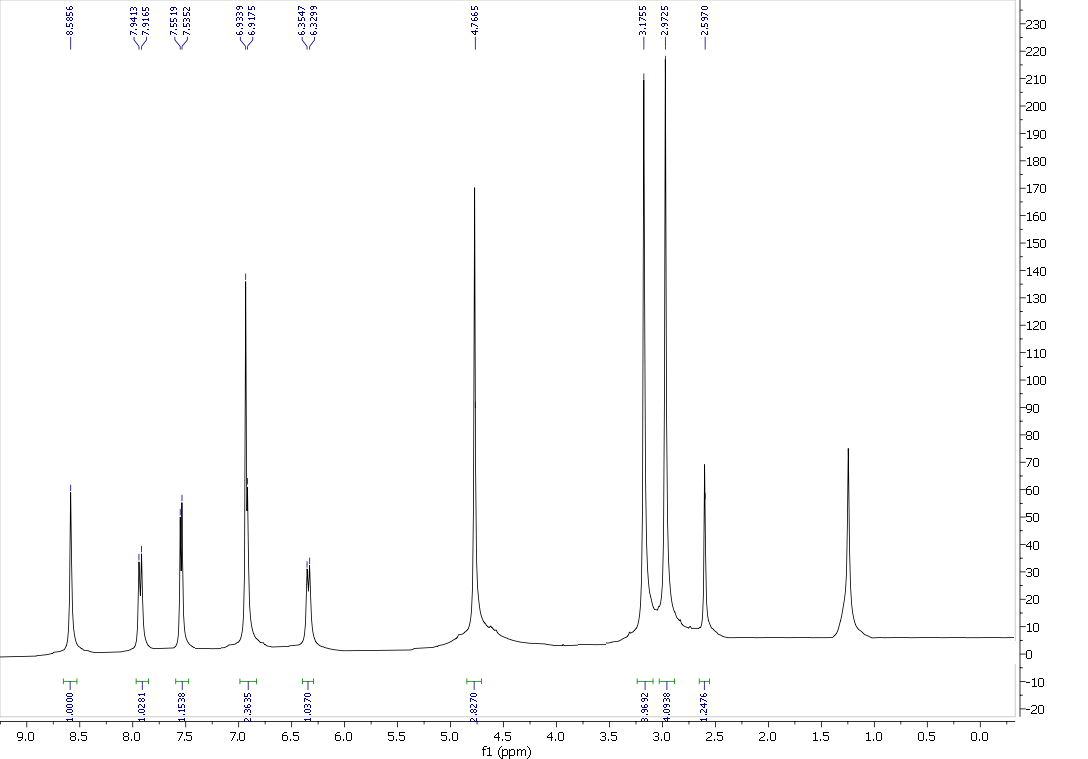


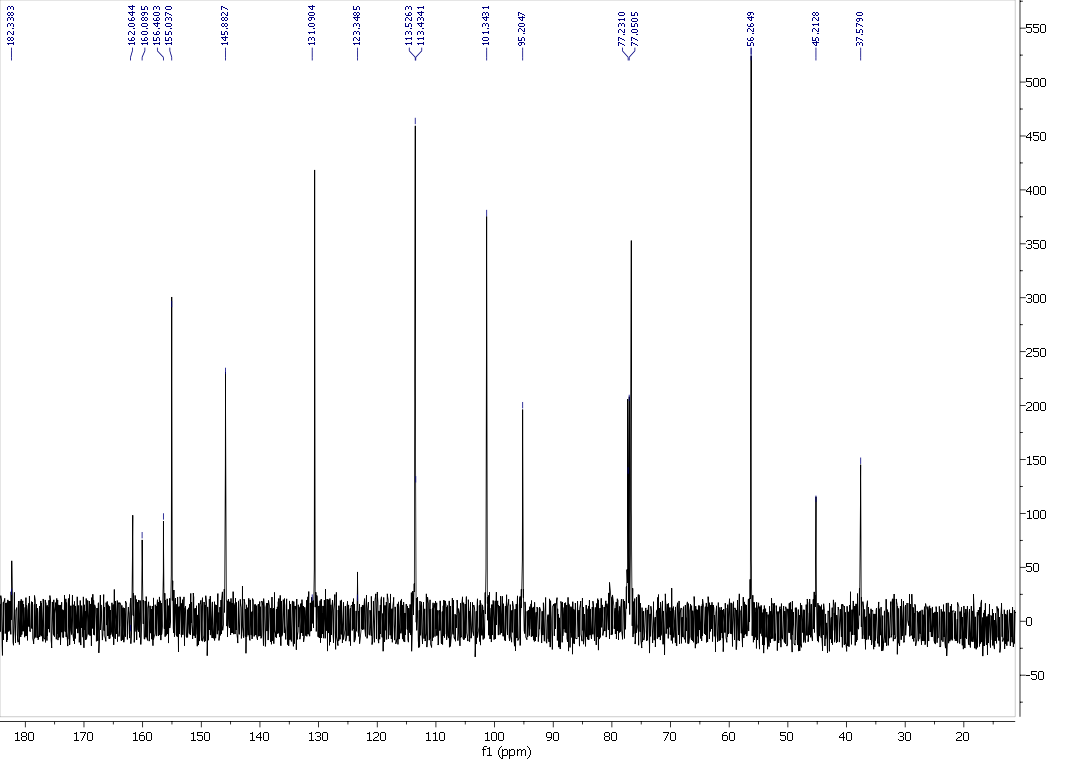


*(E)-7-((1-Benzyl-1H-1,2,3-triazol-4-yl)methoxy)-3-(3-(dimethylamino)acryloyl)-2H-chromen-2-one (****10a****)*

*
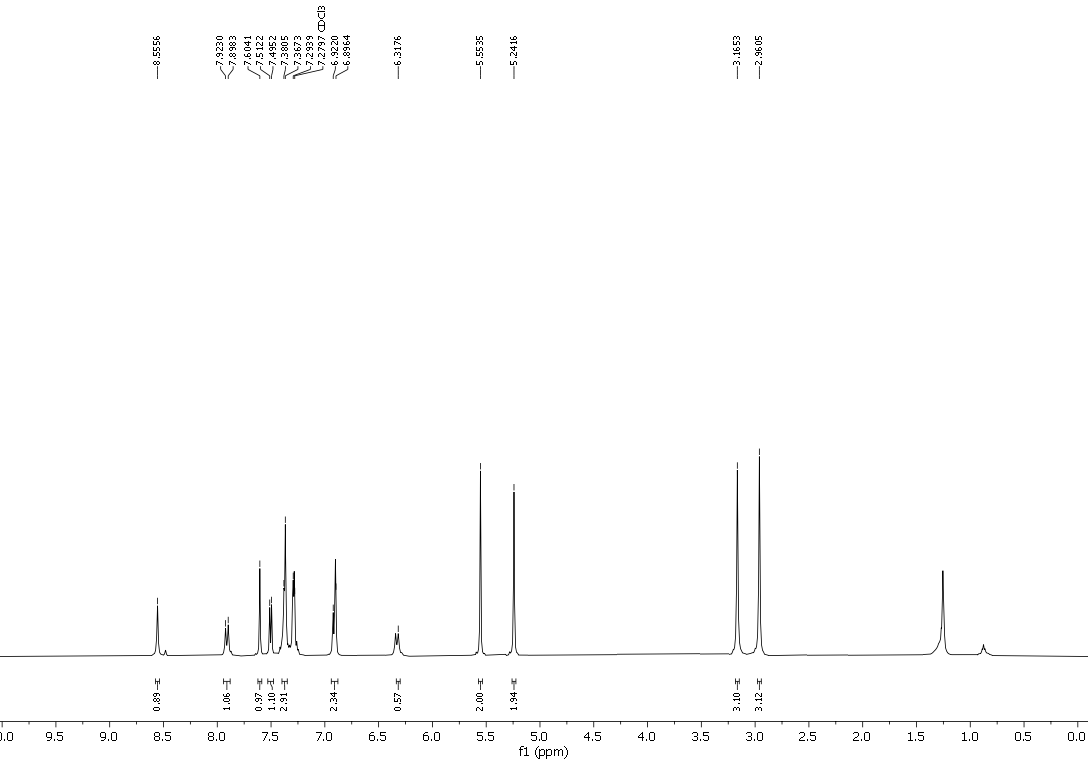
*

*
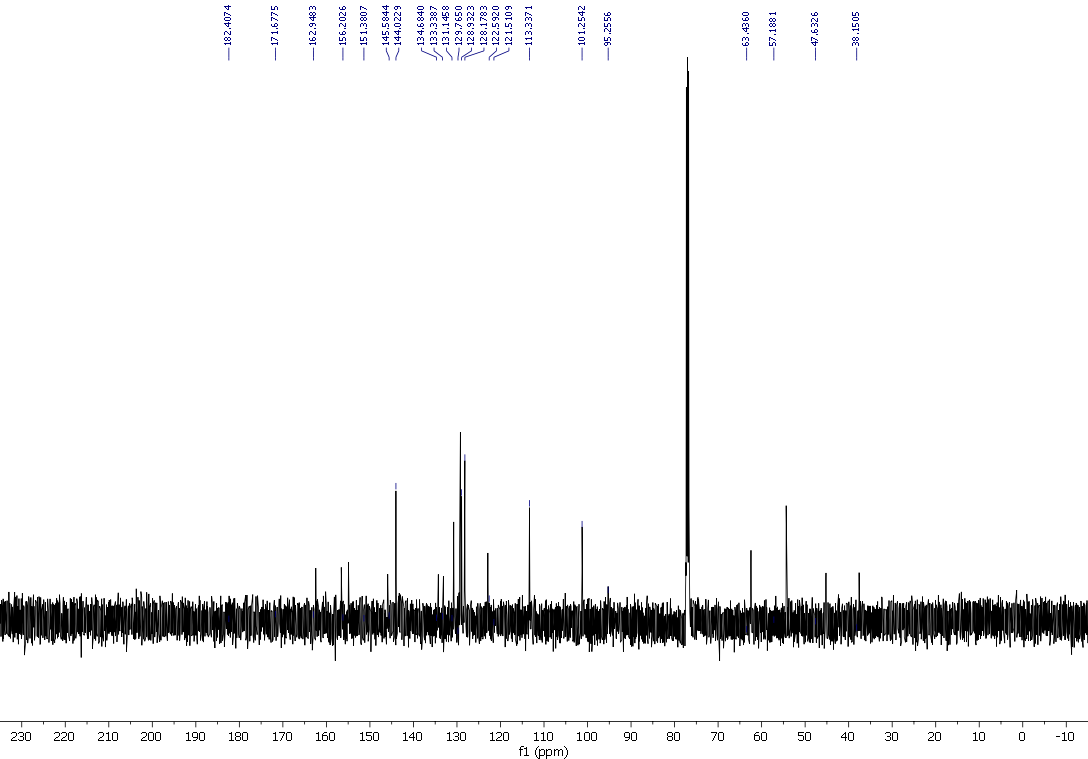
*

*(E)-3-(3-(Dimethylamino)acryloyl)-7-((1-(2-methylbenzyl)-1H-1,2,3-triazol-4-yl)methoxy)-2H-chromen-2-one (****10b****)*


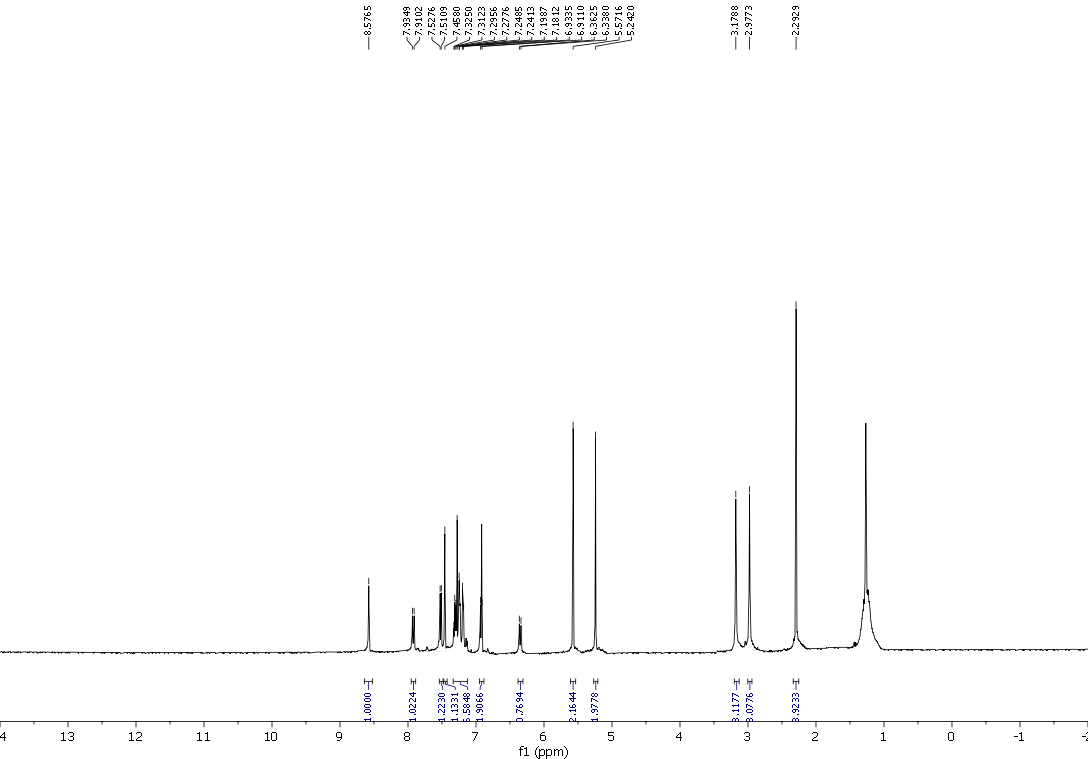


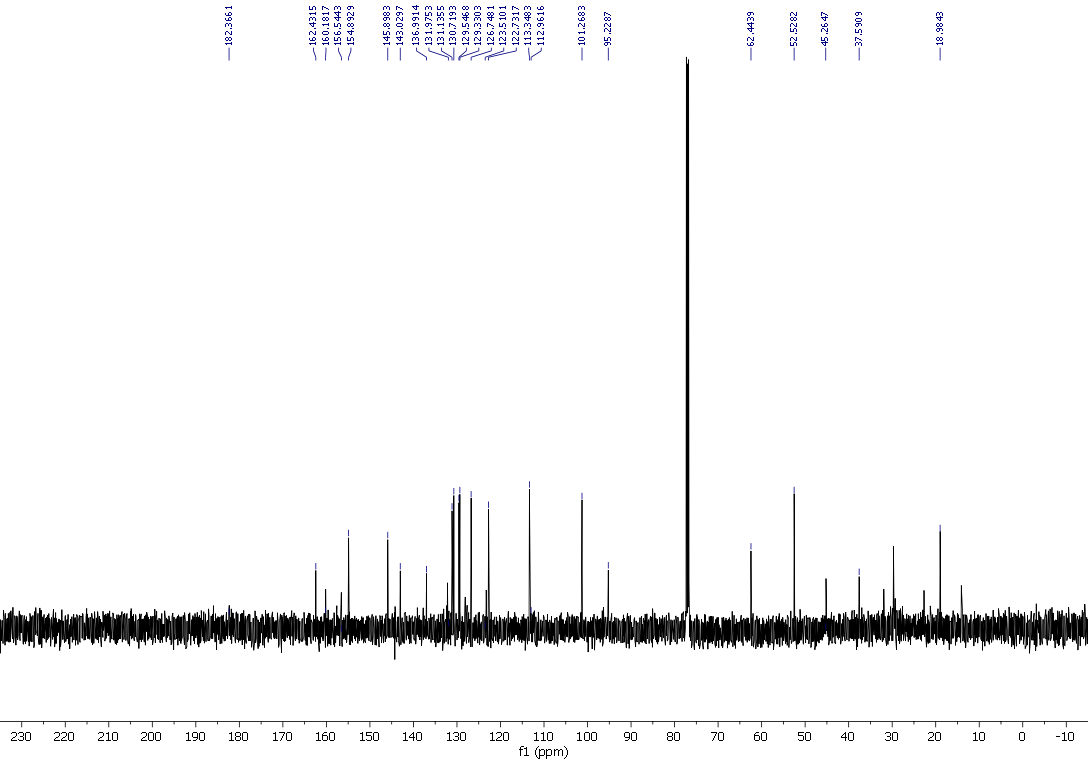


*(E)-3-(3-(Dimethylamino)acryloyl)-7-((1-(4-methylbenzyl)-1H-1,2,3-triazol-4-yl)methoxy)-2H-chromen-2-one (****10c****)*


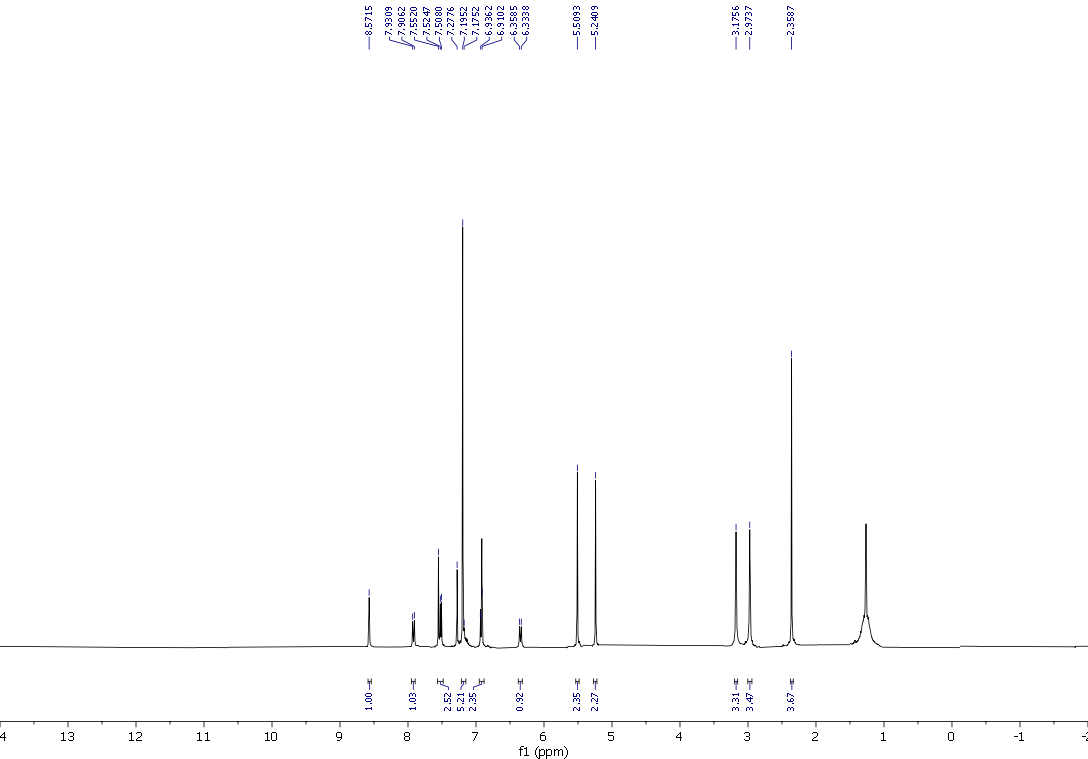


*
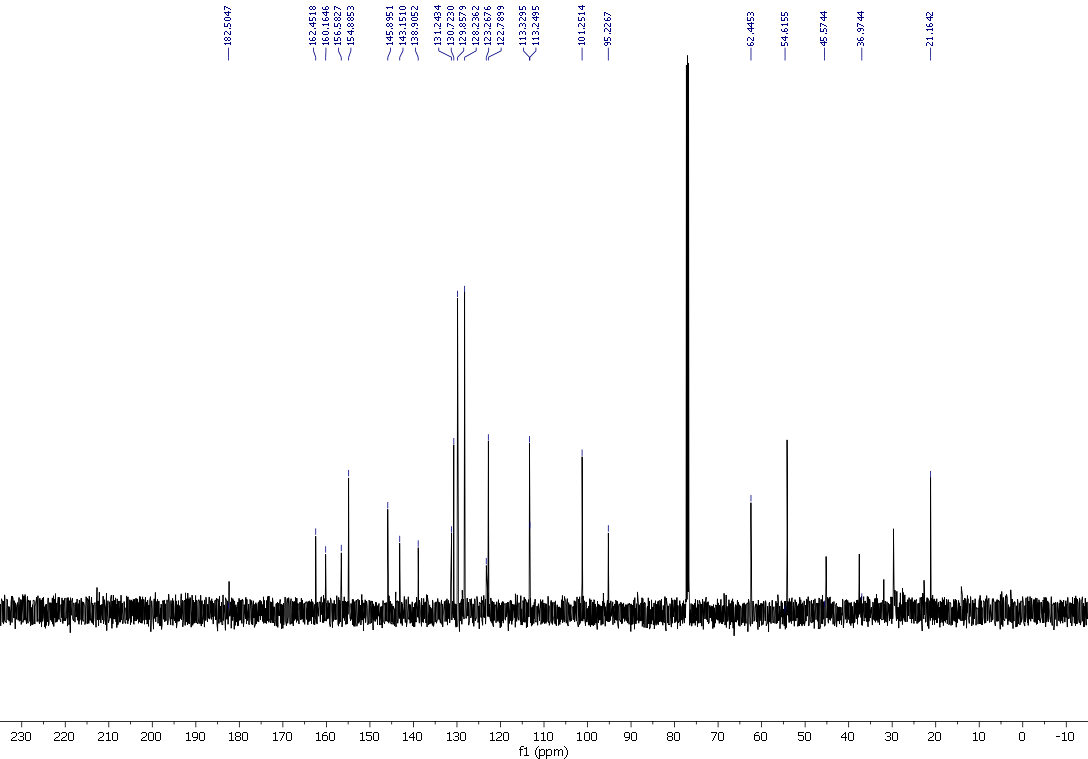
*

*(E)-3-(3-(Dimethylamino)acryloyl)-7-((1-(3-methoxybenzyl)-1H-1,2,3-triazol-4-yl)methoxy)-2H-chromen-2-one (****10d****)*

*
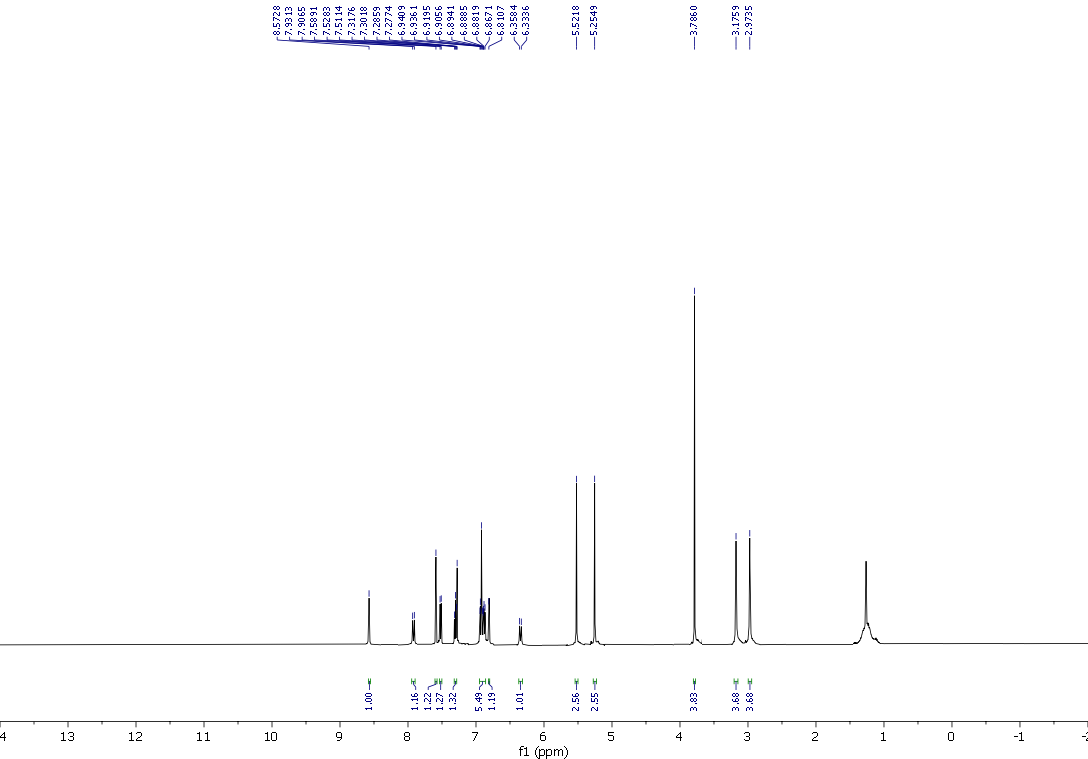
*

*
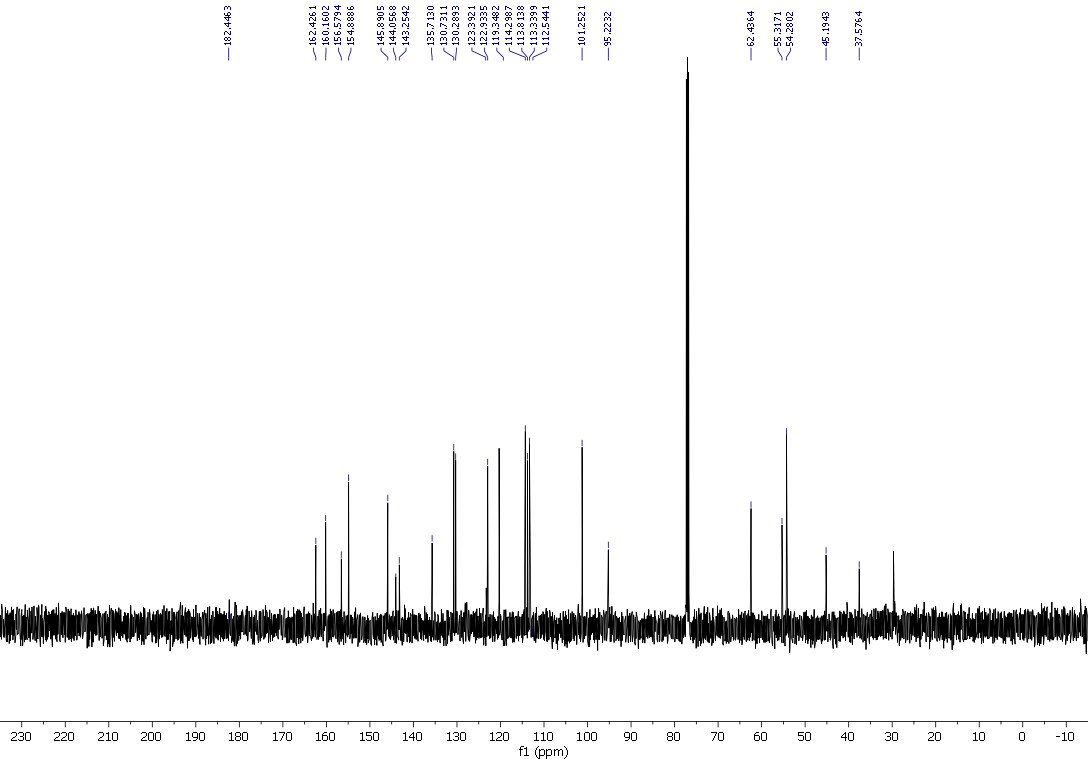
*

*(E)-3-(3-(Dimethylamino)acryloyl)-7-((1-(2-fluorobenzyl)-1H-1,2,3-triazol-4-yl)methoxy)-2H-chromen-2-one (****10e****)*

*
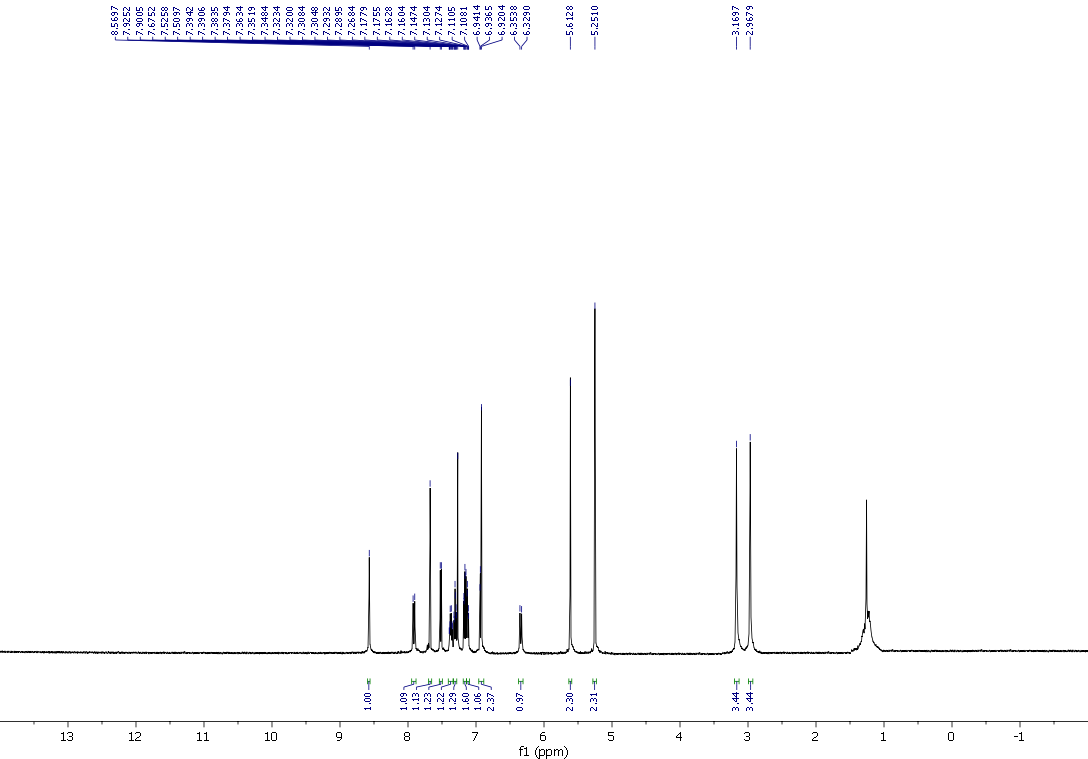
*

*
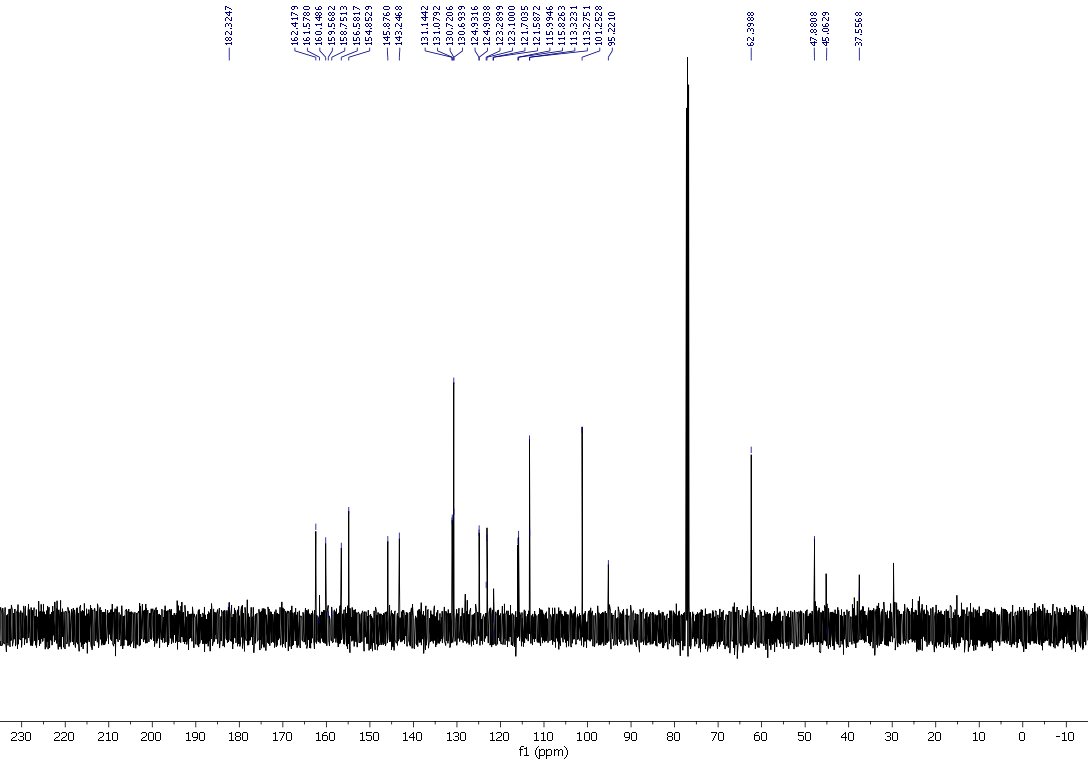
*

*(E)-3-(3-(Dimethylamino)acryloyl)-7-((1-(3-fluorobenzyl)-1H-1,2,3-triazol-4-yl)methoxy)-2H-chromen-2-one (****10f****)*

*
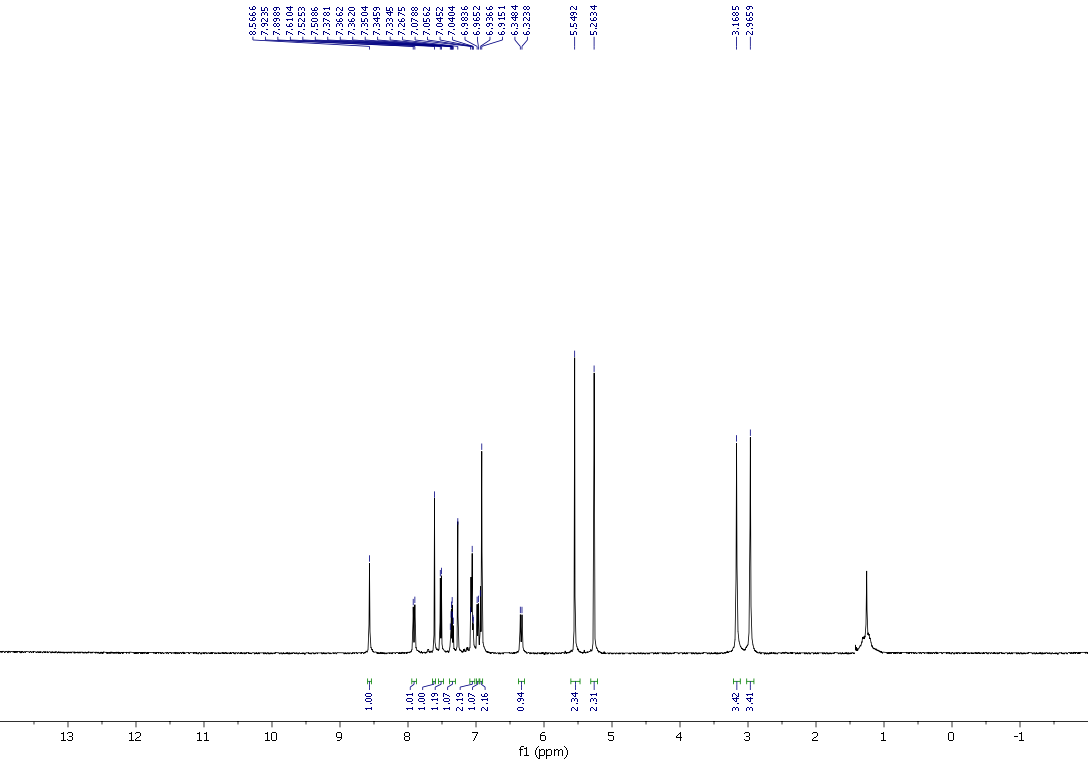
*

*
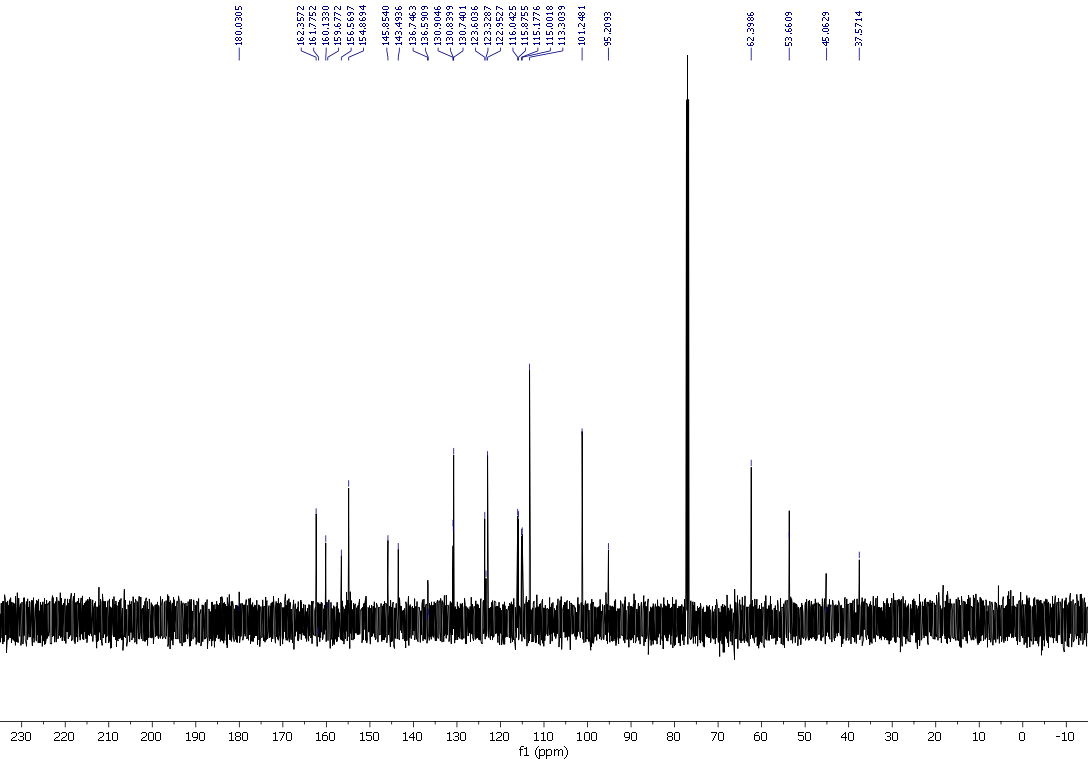
*

*(E)-3-(3-(Dimethylamino)acryloyl)-7-((1-(4-fluorobenzyl)-1H-1,2,3-triazol-4-yl)methoxy)-2H-chromen-2-one (****10g****)*


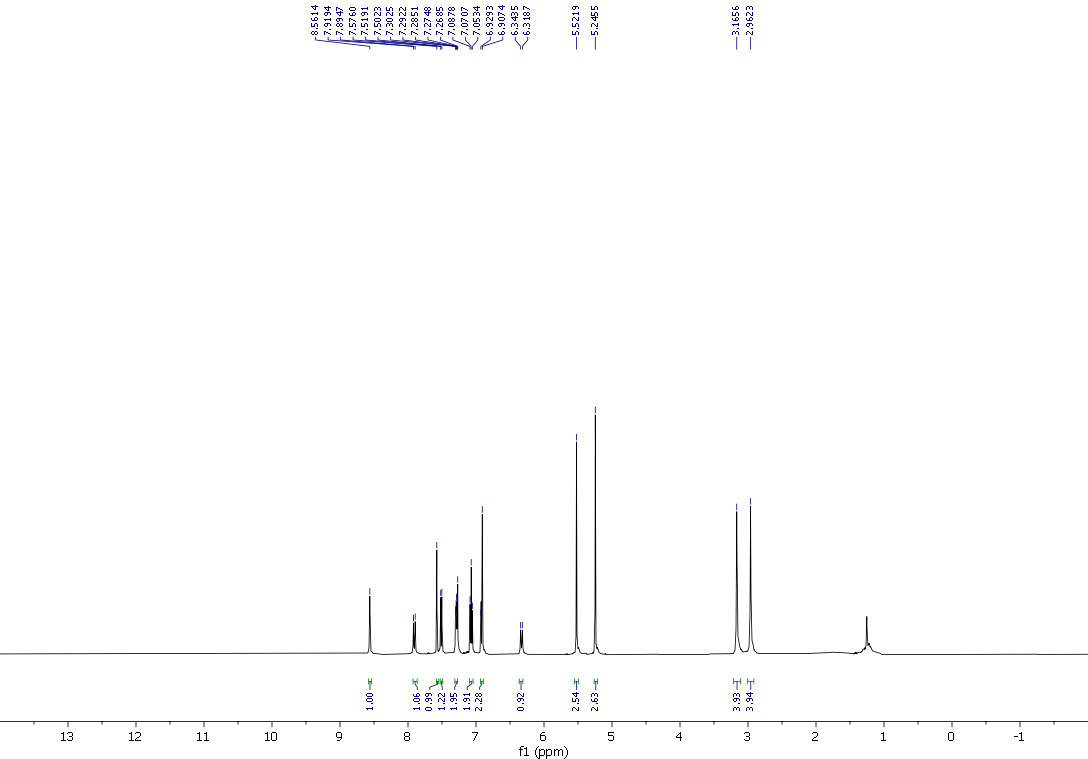


*(E)-7-((1-(3,4-Difluorobenzyl)-1H-1,2,3-triazol-4-yl)methoxy)-3-(3-(dimethylamino)acryloyl)-2H-chromen-2-one (****10h****)*


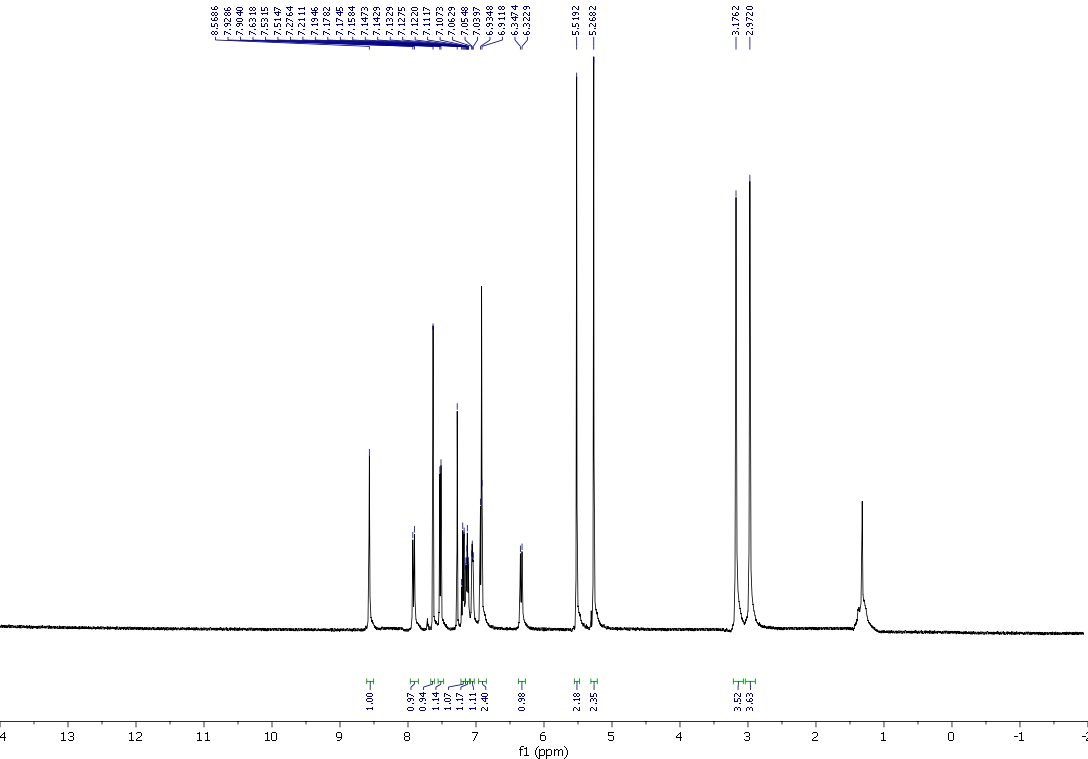


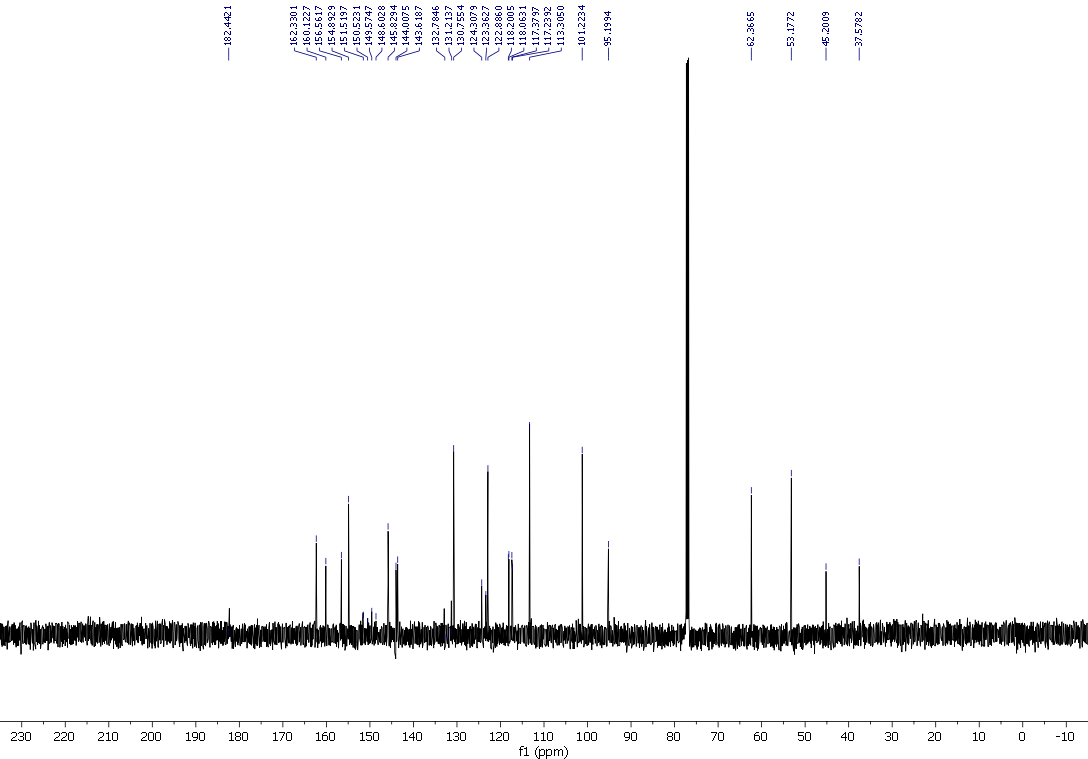


*(E)-7-((1-(2-Chlorobenzyl)-1H-1,2,3-triazol-4-yl)methoxy)-3-(3-(dimethylamino)acryloyl)-2H-chromen-2-one (****10i****)*

*
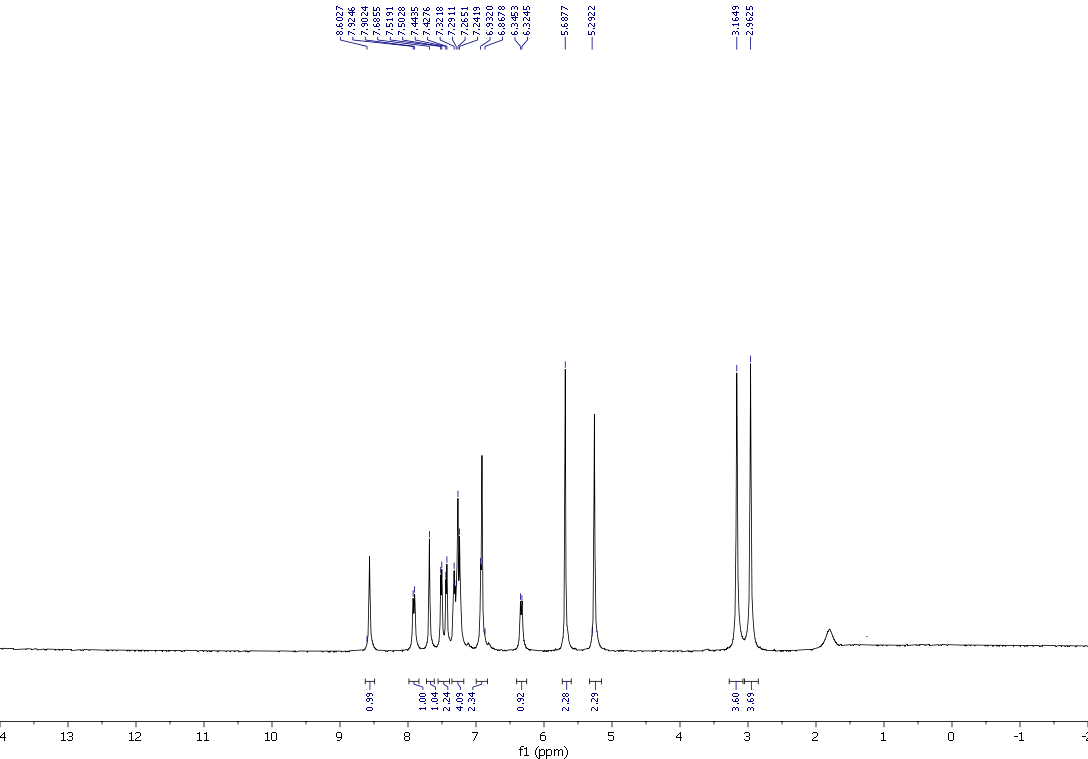
*


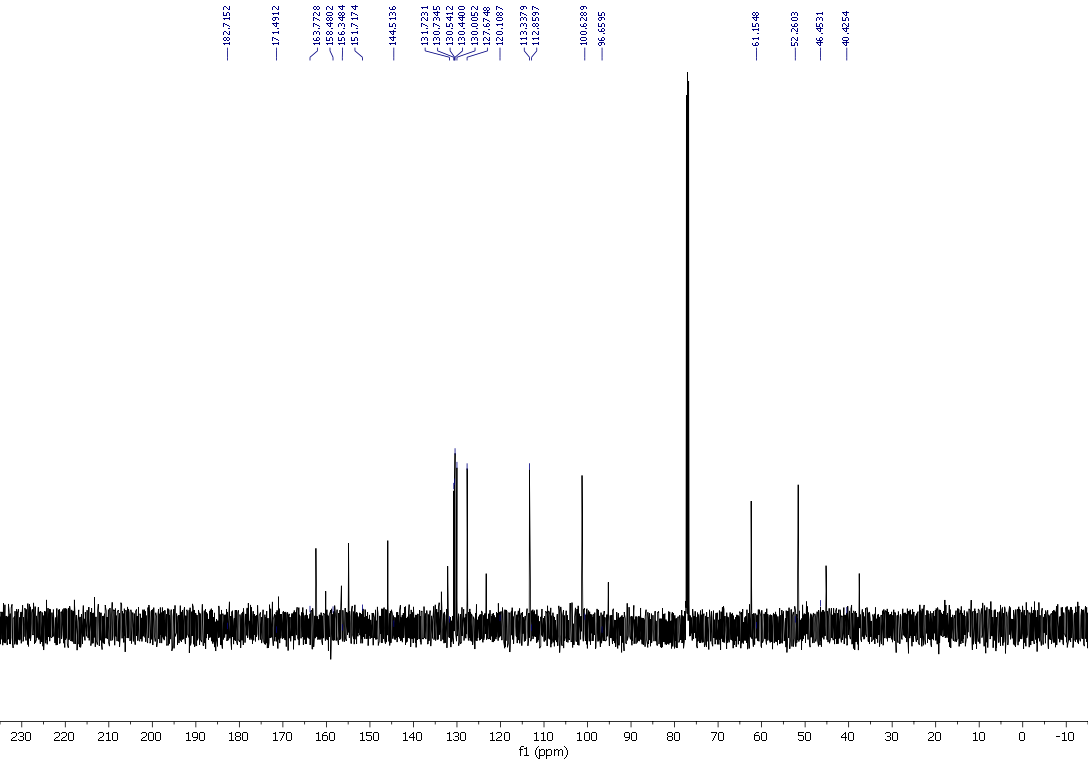


*(E)-7-((1-(4-Chlorobenzyl)-1H-1,2,3-triazol-4-yl)methoxy)-3-(3-(dimethylamino)acryloyl)-2H-chromen-2-one (****10j****)*


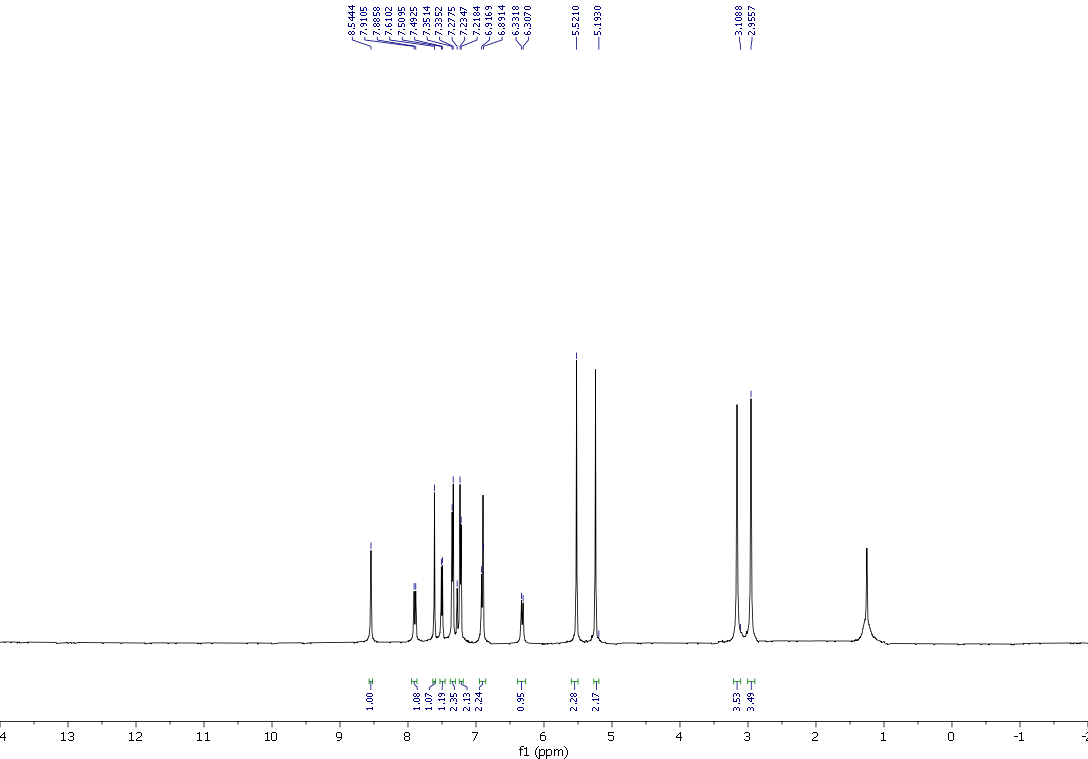


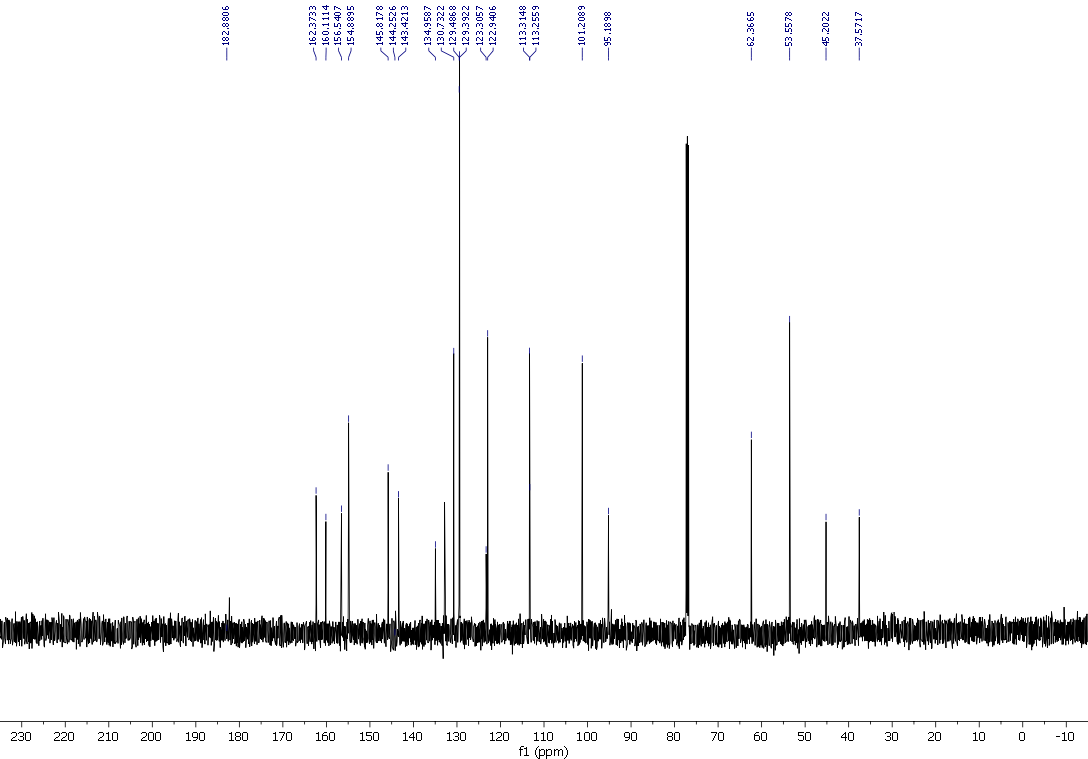


*(E)-7-((1-(2-Bromobenzyl)-1H-1,2,3-triazol-4-yl)methoxy)-3-(3-(dimethylamino)acryloyl)-2H-chromen-2-one (****10k****)*

*
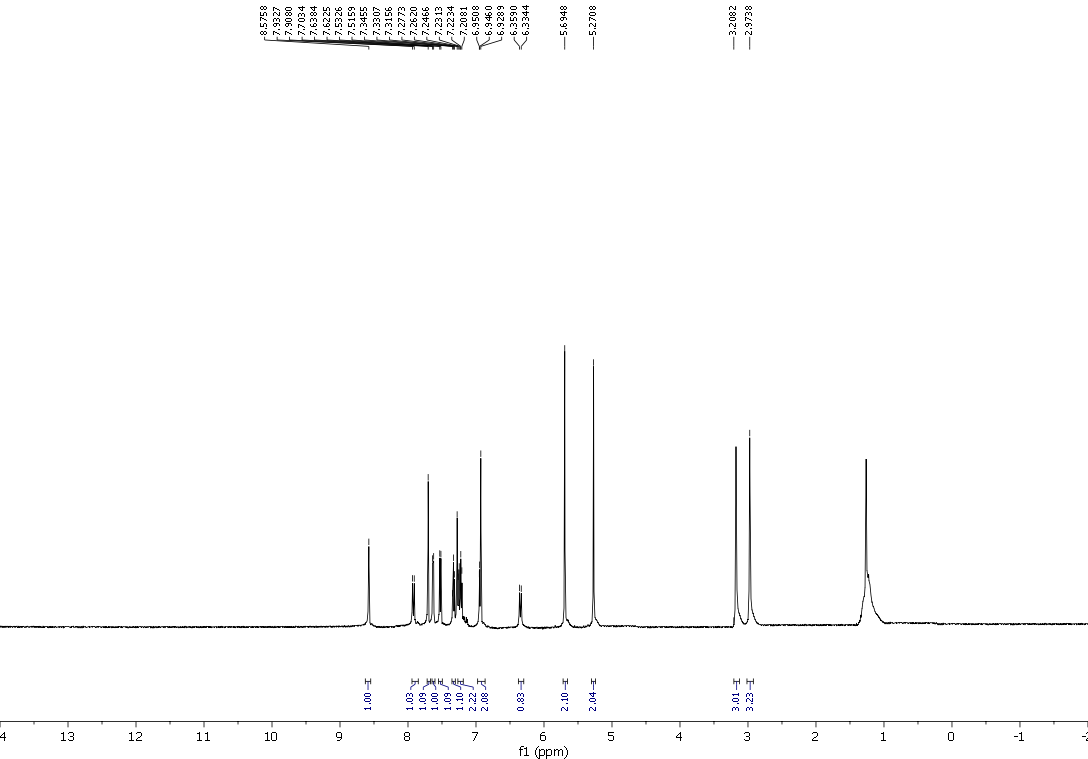
*

*
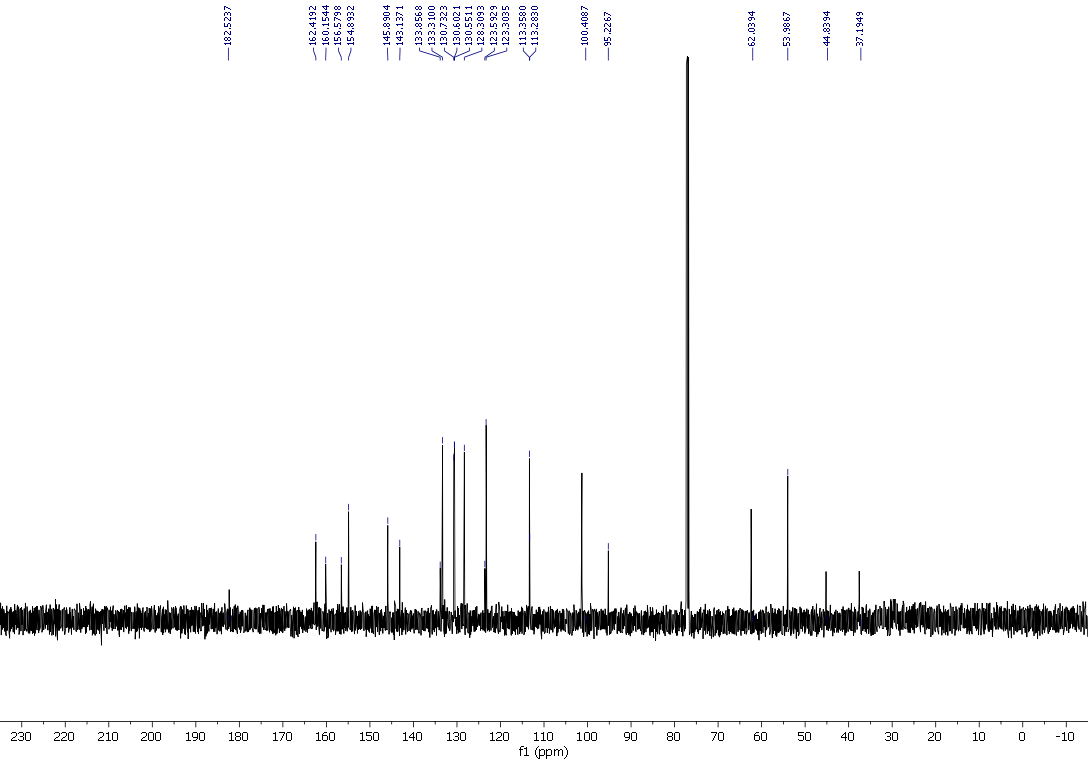
*

*(E)-7-((1-(3-Bromobenzyl)-1H-1,2,3-triazol-4-yl)methoxy)-3-(3-(dimethylamino)acryloyl)-2H-chromen-2-one (****10l****)*


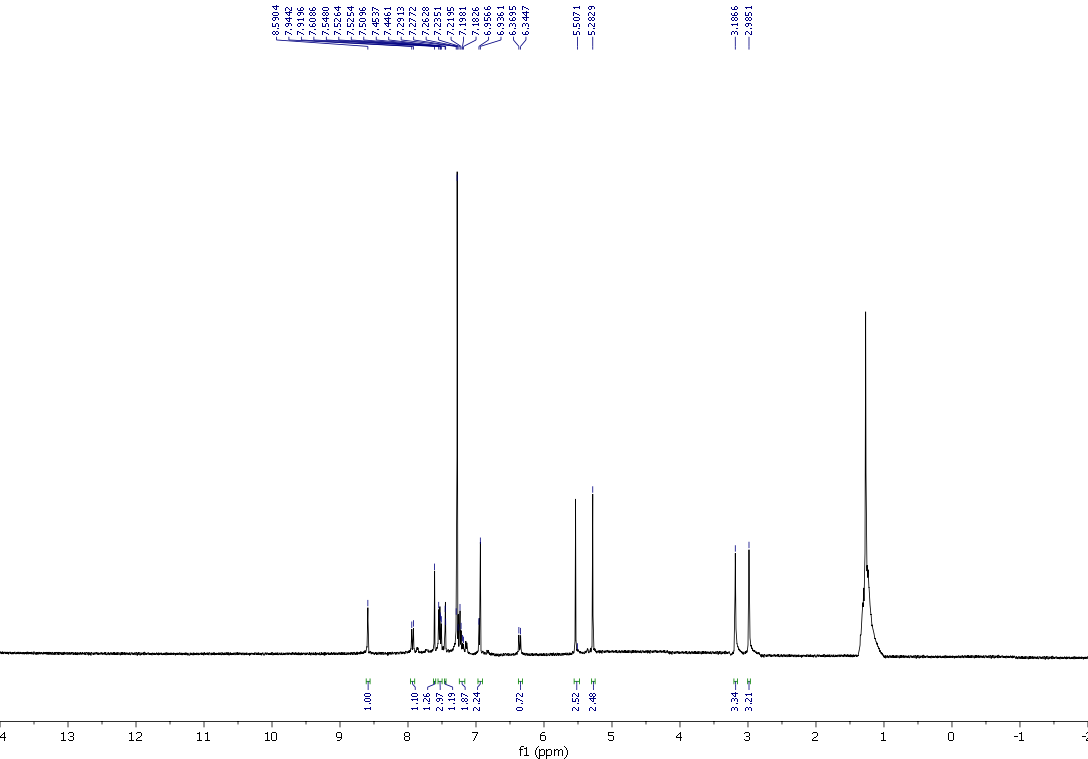


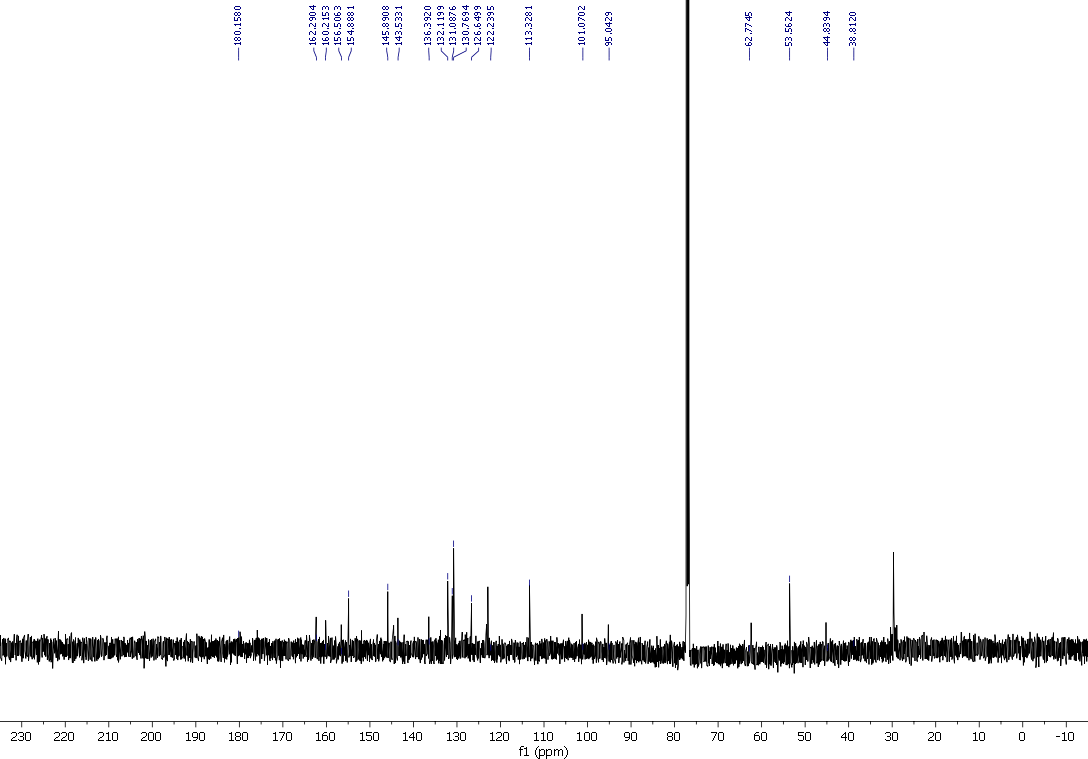


*(E)-7-((1-(4-Bromobenzyl)-1H-1,2,3-triazol-4-yl)methoxy)-3-(3-(dimethylamino)acryloyl)-2H-chromen-2-one (****10m****)*


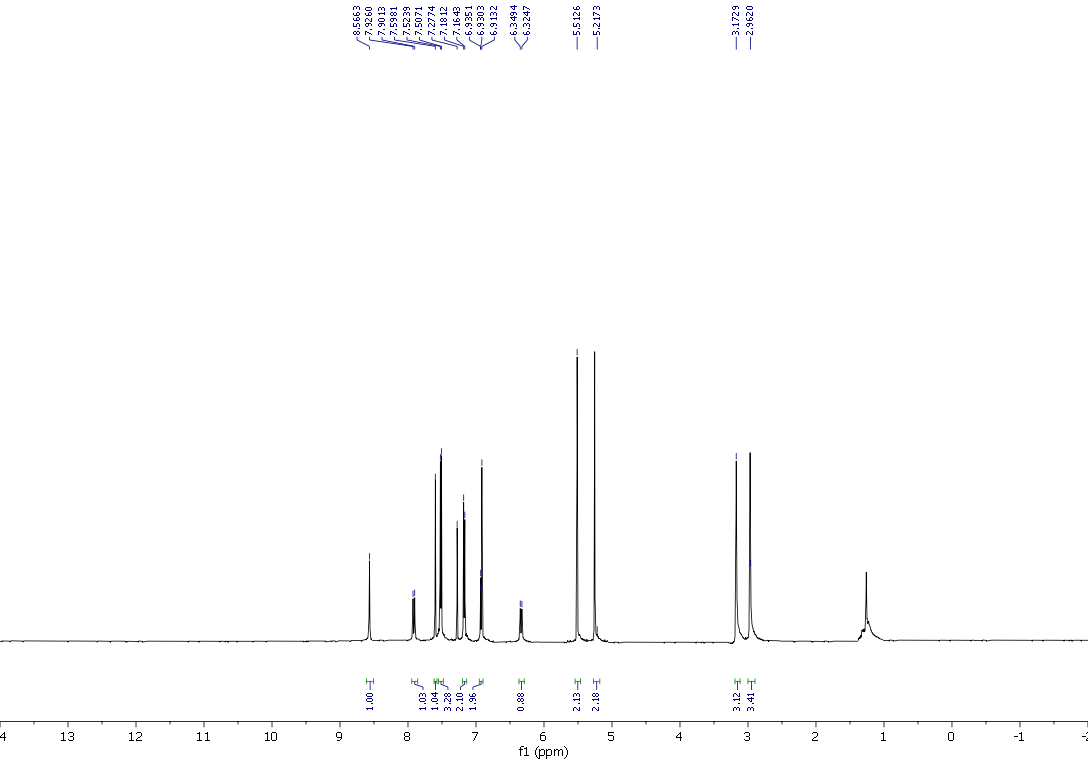


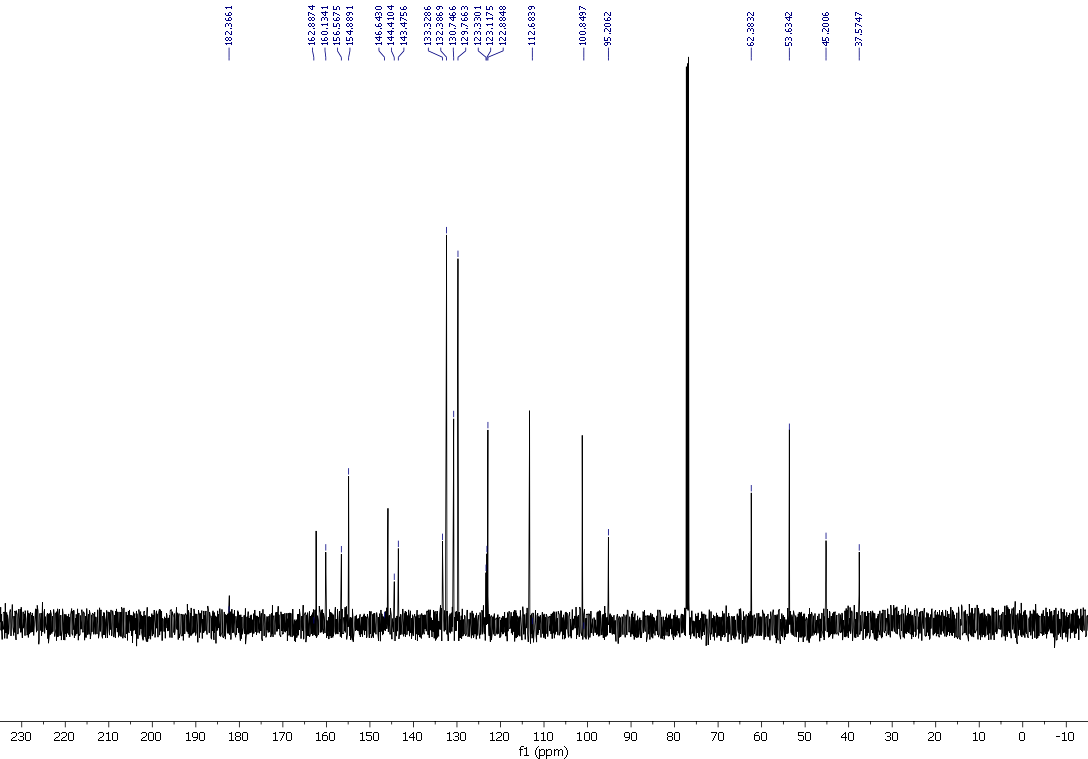


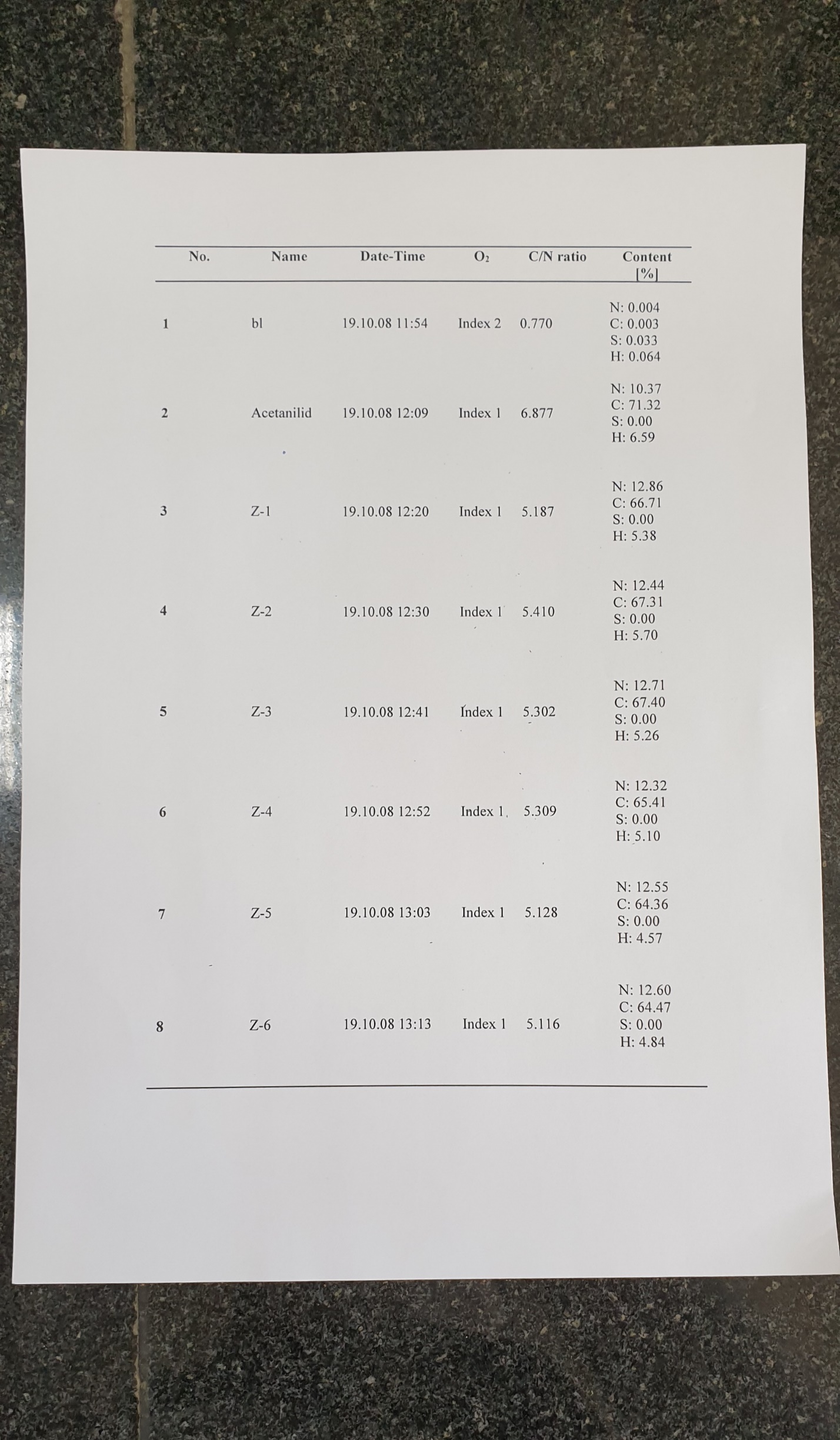


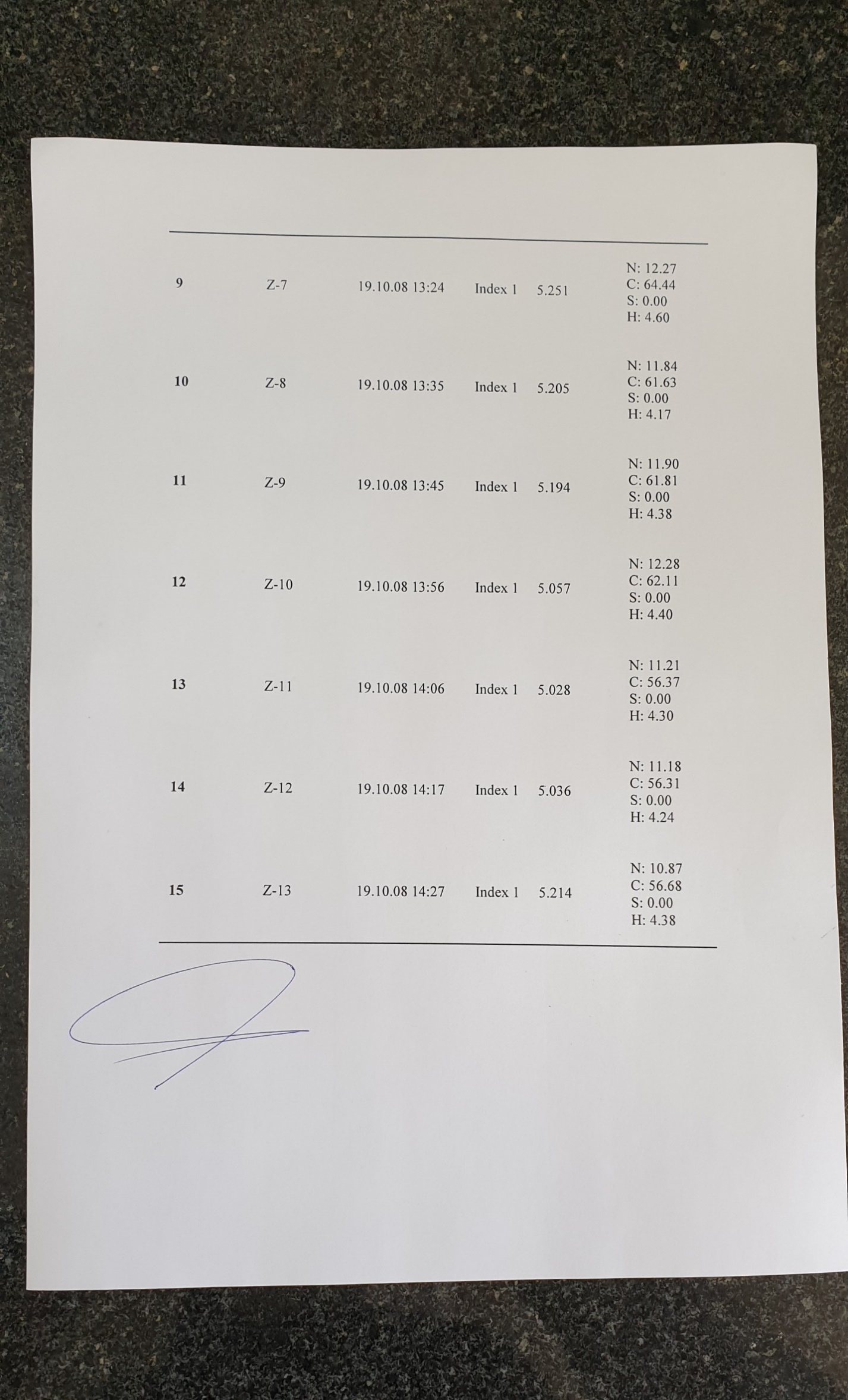

Supplement: Supplementary file 1 — Additional file 1. The supplementary file include copies of NMR spectra and elemental analysis report. [file 13065_2020_715_MOESM1_ESM.docx]
